# Tissues-based chemical profiling and semi-quantitative analysis of bioactive components in the root of *Salvia miltiorrhiza* Bunge by using laser microdissection system combined with UPLC-q-TOF-MS

**DOI:** 10.1186/s13065-016-0187-7

**Published:** 2016-07-13

**Authors:** Wenjian Xie, Hongjie Zhang, Jianguo Zeng, Hubiao Chen, Zhongzhen Zhao, Zhitao Liang

**Affiliations:** School of Chinese Medicine, Hong Kong Baptist University, Kowloon, Hong Kong, Special Administrative Region People’s Republic of China; Hunan Provincial Key Laboratory of Crop Germplasm Innovation and Utilization and National Chinese Medicinal Herbs Hunan Technology Center, Hunan Agricultural University, Changsha, China

**Keywords:** Tanshinones, Salvianolic acids, *Salvia miltiorrhiza* Bunge, Tissues-based analysis, Pharmaceutical quality evaluation

## Abstract

**Background:**

The dry root of *Salvia miltiorrhiza* Bunge (Danshen in Chinese) is an used-widely traditional Chinese herbal medicine with and promising efficacy. This herbal plant has been extensively cultivated in China. Currently, people usually rely on its morphological features to evalaute its pharmaceutical quality. In this study, laser micro-dissection system (LMD) was applied to isolate single fresh tissue of root of *S*. *miltiorrhiza*. Under fluorescent microscopic model, five tissues namely cork, cortex, phloem, xylem ray and vessel were well recognized and isolated accurately by LMD, respectively and then the distribution pattern of the major bioactive compounds in various tissues was investigated by ultra-performance liquid chromatography-quadrupole/time of flight-mass spectrometry, which aims to validate the traditional experience on evaluating pharmaceutical quality of Danshen by morphological features.

**Results:**

Total 62 chemical peak signals were captured and 58 compounds including 33 tanshinones, 23 salvianolic acids and 2 others were identified or tentatively characterized in micro-dissection tissues. Further semi-quantitative analysis indicated that the bioactive components such as tanshinones and salvianolic acids were mainly enriched in cork tissue.

**Conclusion:**

In the present study, analysis of metabolic profile in different tissues of roots of *S*. *miltiorrhiza* is reported for the first time. The distribution pattern of major bioactive components could enable medicinal vendors and consumers to relatively determine the pharmaceutical quality of Danshen by morphological features.

**Graphical abstract:**
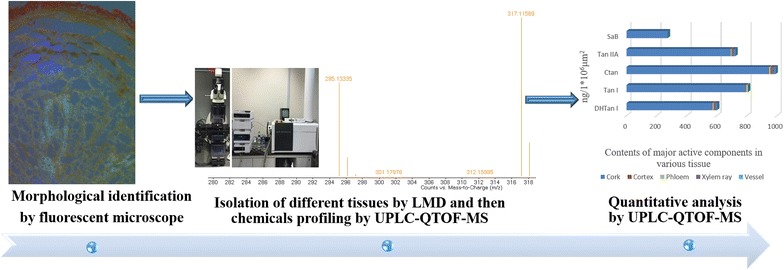
Tissues-based chemical profiling of Danshen

**Electronic supplementary material:**

The online version of this article (doi:10.1186/s13065-016-0187-7) contains supplementary material, which is available to authorized users.

## Background

The dry root of *Salvia miltiorrhiza* Bunge, namely Danshen in Chinese, which is an important traditional Chinese herbal medicine. Over two thousand years ago, Danshen has been categorized as a superior grade herbal medicine by The Divine Husbandman’s Classic of Materia Medica (Shen Nong Ben Cao Jing), which means that it can be beneficial to human’s health and it is safe, even it is taken for a long time [1]. Today, it has been used as a principal drug in many proprietary Chinese medicines for treating coronary heart disease, cerebrovascular disease, irregular menstruation and hepatosplenomegaly [2]. Around 20 kinds of proprietary Chinese medicines such as compound Danshen capsules, compound Danshen tablets, Danshen injection and compound Danshen dripping pills (CDDP) have been developed and some of its relative products have also been used as over the counter medicine (OTC) in Japan [3, 4]. Moreover, CDDP has been approved to carry out phase III clinical trial for preventing and treating stable angina and diabetic retinopathy by U.S. FDA [5].

Due to the increasing demands of this plant resources and extensive application in clinic, *S. miltiorrhiza* has been widely cultivated in Sichuan, Shangxi, Shanxi, Henan, Hebei, Shandong, Anhui, Hubei, Jiangsu and Zhejiang provinces of China and the supply of Danshen has been dominated by cultivated resource. According to the traditional experiences on morphological evaluation and classification of Danshen, it is divided into different grades by their size of main root and the color of outer bark for better transaction in the commercial markets [3]. As we know, however, the pharmaceutical quality of herbal medicines may be easily affected by some factors such as producing areas, harvest season and even cultivation technologies. Up to now, no objective evidences have been found to prove that the bigger size of main root and deeper brown–red of appearance of this medicinal plant could indicate the better pharmaceutical quality. It is no doubt that it is still unclear whether such simple quality classification criteria can really reflect its pharmaceutical quality or not. In addition, for quality evaluation of Danshen, although modern chromatographic methods involving HPLC fingerprint and determination of main components by HPLC have been established [4, 6], it is hard for medicinal vendors and consumers to equip with modern instruments to evaluate the quality of Danshen. On the other hand, it is well known that evaluating the quality of various grades of Chinese herbal medicines by morphological features is a convenient, quick and practical method compared with other methods that mostly rely on modern instruments.

Several pharmacological studies have demonstrated that bioactive effects of Danshen are mainly attributed to its secondary metabolites including diterpene quinones and salvianolic acids such as tanshinone I (Tan I), dihydrotanshinone I (DHTan I), tanshinone II_A_ (Tan II_A_), cryphtotanshinone (CTan) and salvianolic acid B (SaB) [7–9]. Mapping the distribution of these bioactive components and carrying out semi-quantitative analysis in various herbal tissues can help to evaluate pharmaceutical quality of herbal medicine. Laser micro-dissected system (LMD) plus with ultra-performance liquid chromatography-quadrupole-time of flight-mass spectrometry (UPLC-Q-TOF-MS) has been demonstrated as a powerful tool to establish an objective relationship between major bioactive second metabolites and morphological features of herbal medicine [10–13]. Here, this strategy was firstly applied to validate the traditional experience and judge them as true or false views, with regard to pharmaceutical quality, which is important for the quality evaluation and classification of different grades of Danshen.

## Experiment section

### Plant materials

The plant materials (Table 1) were collected from eight cultivation bases and one natural habitat in China. All of them were authenticated as *S. miltiorrhiza* Bunge by Dr. Zhitao Liang from school of Chinese Medicine, Hong Kong Baptist University and the specimens were deposited in the Bank of China (Hong Kong) Chinese Medicines Centre of Hong Kong Baptist University.

**Table 1:** Sample information of *S. miltiorrhiza* in the present study

Sample no.	Colour of outer bark^a^	Size^b^ (cm)	Sources	Collection date
S1	Brownish–red	0.8	Cultivation, Zhongjiang County, Sichuan province	2014.11.19
S2	Dark brownish–red	1.3	Cultivation, Shangluo City, Shanxi province	2014.11.19
S3	Dark brownish–red	0.75	Cultivation, Fangcheng County, Henan province	2014.11.19
S4	Brownish–red	1.4	Wild, Henan province	2014.11.19
S5	Brownish–red	0.7	Cultivation, Linqu County, Shandong province	2014.11.19
S6	Brownish–red	1.0	Cultivation, Beijing	2014.11.19
S7	Brownish–red	0.5	Cultivation, Beijing	2014.11.19
S8	Brownish–red	0.65	Cultivation, Beijing	2014.11.19
S9	Brownish–red	1.0	Cultivation, Nanjing, Jiangsu province	2015.05.31

^a^Colour of outer bark refer to Fig. 6

^b^Size calculated by diameter of main root of *S. miltiorrhiza*

### Chemicals and reagents

**Fig. 1:**
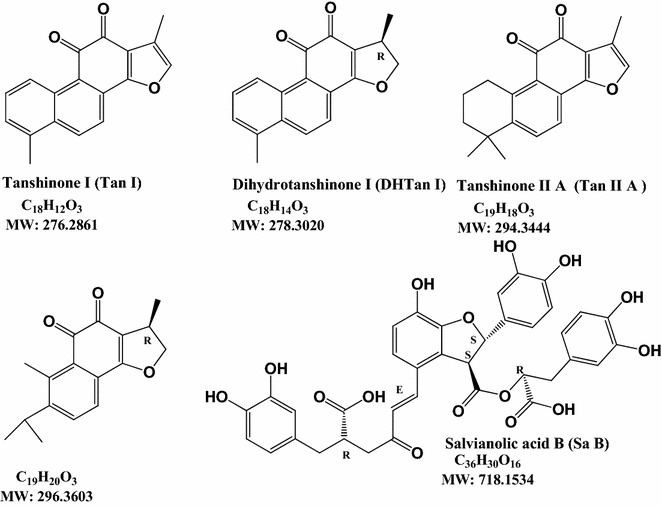
Chemical structures of 5 chemical markers

Chemical markers including Tan I, DHTan I, Tan II, CTan and Sa B were purchased from Chengdu Must Bio-Technology Co., Ltd. (Chengdu, People’s Republic of China) (Fig. 1). The purity of each standard was over 98 %. Both acetonitrile and methanol (HPLC grade) were purchased from E. Merck (Darmstadt, Germany) and formic acid (HPLC grade) was ordered from Tedia, USA. Water for analyzing was prepared by a Mili-Q water purification system (Millipore, Bedford, MA, USA).

### Materials and instruments

Cryotome (Thermo Shandon As620 Cryotome, Cheshire, UK), Cryogen (Thermo Shandon, Cheshire, UK), Non-fluorescent polyethylene terephthalate (PET) microscope steel frame slide (76 × 26 mm, 1.4 μm, Leica Microsystems, Bensheim, Germany), Leica Laser microdissection 7000 system, 500 μL micro-centrifuge tube (Leica), Centrifuge (Centrifuge 5417R, Eppendorf, Hamburg, Germany), Ultrasonic instrument (CREST 1875HTAG Ultrasonic Processor, CREST, Trenton, NJ), HPLC grade vial (1.5 mL, Grace, Hong Kong), Glass-lined pipe with plastic ring (400 μL, Grace, Hong Kong), Electronic balance (Mettler Toledo MT5 style), Agilent 6540 ultra-definition accurate mass quadrupole time-of-flight spectrometer equipped with a mass hunter workstation software (Agilent version B.06.00 series, Agilent Technologies, USA), Acquity UPLC BEH C_18_ column (2.1 mm × 100 mm, 1.7 μm) coupled with a C_18_ pre-column (2.1 mm × 5 mm, 1.7 μm, Waters, USA).

### Samples preparation

The protocol of samples preparation for analysis was usually divided into three stages. Firstly, each prepared fresh root was fixed by cryogen and frozen on a −35 °C cryo bar, before being cut into 30 μm cross-section of tissue and attached on a non-fluorescent polyethylene terephthalate. At the next stage, each prepared cross-section of tissue was exposed to a Leica LMD-BGR fluorescence filter system at 6.3 magnification for microscopic authentication (field of 6, color saturation of 1.20, exposure time of 777 μs, gain of 2.5, and IFW1 light intensity of green and diaphragm of 5), after then 5 different target tissue, around 1 × 10^6^ μm^2^ per each (Table 2), were individually isolated by Laser Micro-dissection system (7000 V 7.5.0.5112 edition) with an optimal parameters (DPSS laser bean wavelength of 349 nm, power of 53 μJ, aperture of 46, speed of 2, specimen balance of 41, head current of 100 %, plus frequency of 4046 Hz), before collecting it by a cap of 500 μL micro-centrifuge tube. Finally, each prepared sample was sent to centrifuge 5 min (12,000 rpm, 20 °C) in order to ensure it fell into the bottom from the cap, and then added 100 μL methanol into each micro centrifuge tube for ultrasonic extraction 60 min and then centrifuged again 10 min (12,000 rpm, 20 °C). 90 μL supernatant was transferred into a glass-lined pipe with a plastic ring accommodated by a HPLC grade vial and stored at a 4 °C refrigerator.

**Table 2:** Total micro-dissected area in different tissues

Sample no.	Special tissue/total micro-dissected area (μm^2^)				
	Cork	Cortex	Phloem	Xylem ray	Vessel
S1	1,006,611	1,003,330	1,063,204	1,022,559	1,020,931
S2	1,000,990	1,000,072	1,000,320	1,000,791	1,000,276
S3	1,000,160	1,003,816	1,000,051	1,000,830	1,000,686
S4	1,000,011	1,000,962	1,000,249	1,000,343	1,000,589
S5	1,000,583	1,000,699	1,000,983	1,000,300	1,000,349
S6	1,000,736	1,001,599	1,000,860	1,001,172	1,000,058
S7	1,003,180	1,000,194	1,000,901	1,000,148	1,001,122
S8	1,000,609	1,000,606	1,000,728	1,000,407	1,000,354
S9	1,000,402	1,000,365	1,000,629	1,000,310	1,000,291

### Standard solution preparation

Each standard including Tan I, DHTan I, Tan II_A_, CTan and Sa B was accurately weighed and dissolved individually in methanol to produce mixed stock solution with concentrations at 0.96 mg/mL of Sa B, 0.992 mg/mL of DHTan I, 0.954 mg/mL of Tan I, 0.991 mg/mL of CTan, 1.028 mg/mL of Tan II_A_. The series concentrations of mixed working solution were prepared by diluting the mixed stock solution with methanol. In addition, due to the high sensitive requirement in UPLC-QTOF-MS, here a blank control containing solvent was set to exclude the negative impact on analyzing process.

### Method of UPLC-QTOF-MS

According to the results of preliminary experiment, the optimal running parameters of UPLC were set as follows: the mobile phase consisted of water with 0.1 % formic acid (A) and acetonitrile with 0.1 % formic acid (B) with an procedure of linear gradient elution: 0-8 min (40 % B), 8–20 min (40–75 % B), 20–22 min (75–100 % B), 23–25 min (100 % B), the injection volume was 3 μL and the flow rate was set at 0.35 mL/min. Salvianolic acids were more sensitive in negative ion scanning mode while tanshinones were more sensitive in positive ion scanning mode, so the mass spectra were acquired in both positive and negative modes by scanning from 100 to 1700 in mass to charge ratio (m/z), the scanning of MS was performed under the following operation parameters: dry gas temperature of 325 °C, dry gas (N_2_) flow rate of 8 L/min, nebulizer pressure of 45 psi, V-cap of 4500, nozzle voltage 500 V, and fragmentor 150 V.

## Results and discussion

### Microscopic characteristics and separation of tissues

**Fig. 2:**
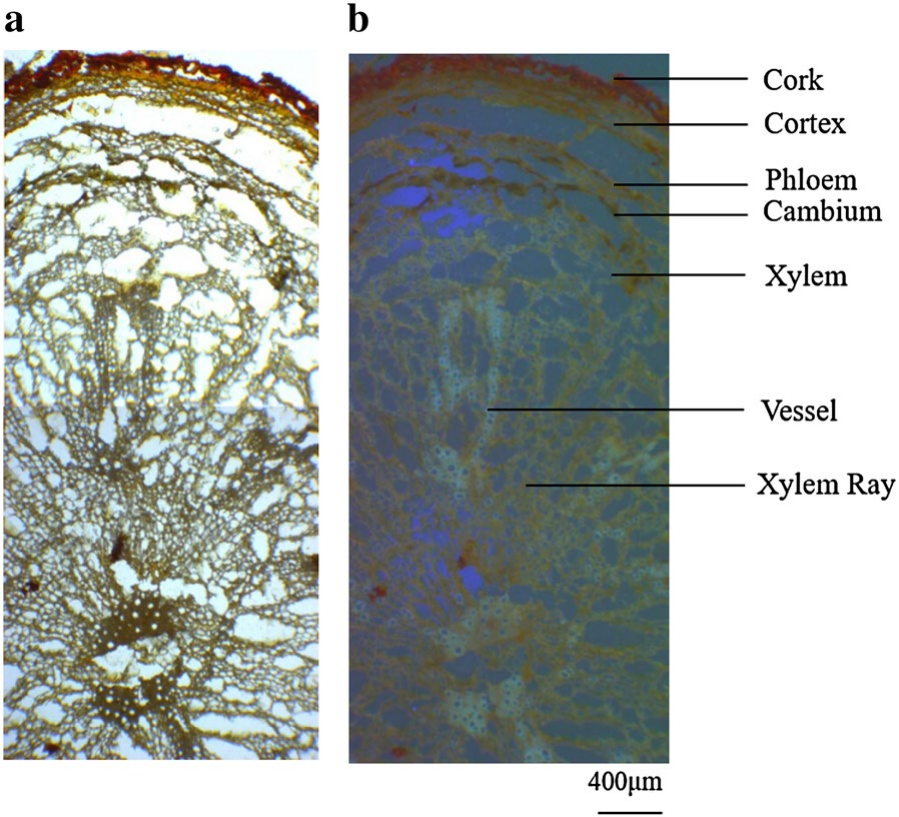
Cross-sections of the root of *S. miltiorrhiza* (S7) **a** observed under the bright filed mode **b** observed under the fluorescent mode

Here, sample seven was used as a representative to present the microscopic characteristics of whole cross-section of root observed under bright filed and fluorescence mode (Fig. 2). Under the bright filed, the anatomical features of root were found to be mainly composed of cork, cortex, phloem, cambium, xylem ray and vessel (from external to internal part). Cork was brownish–red and consisted of several layers of narrow cells and cortex showed brownish–yellow color and lied with several layers of fat cells. The boundary between phloem and cambium was unclear. Wide xylem ray were found at the middle between each two grouped or single vessels. When observed by fluorescence mode, cork also showed similar color as observed in bright filed. Cortex exhibited brownish–yellow. Phloem and xylem showed the similar fluorescence while vessels showed yellowish–white. According to structural characteristics of tissues under fluorescence mode, fives tissues namely cork, cortex, phloem, xylem ray and vessels were isolated for analyzing, respectively.

### Identification of chemicals in various tissues

**Fig. 3:**
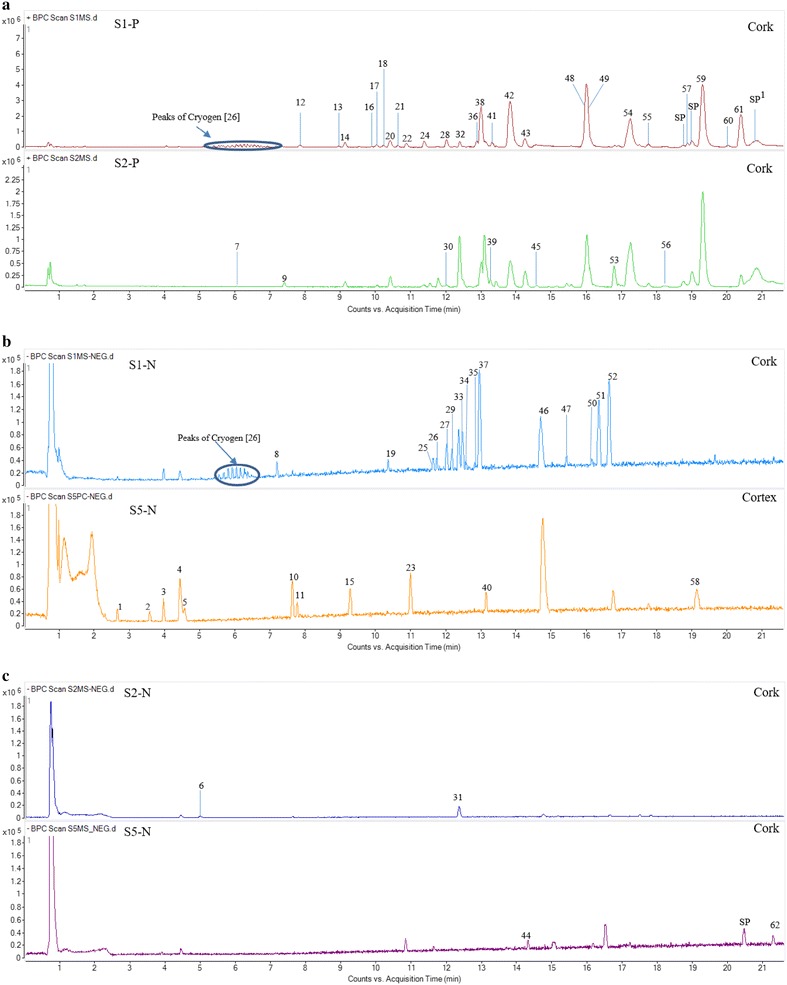
The represent BPC chromatograms from cork tissue of S1 and S2 detected under positive mode (**a**), cork tissue of S1 and cortex tissue of S5 (**b**) as well as cork tissue of S2 and S5 (**c**) detected under negative mode.^1^ *SP* solvent peak

**Fig. 4:**
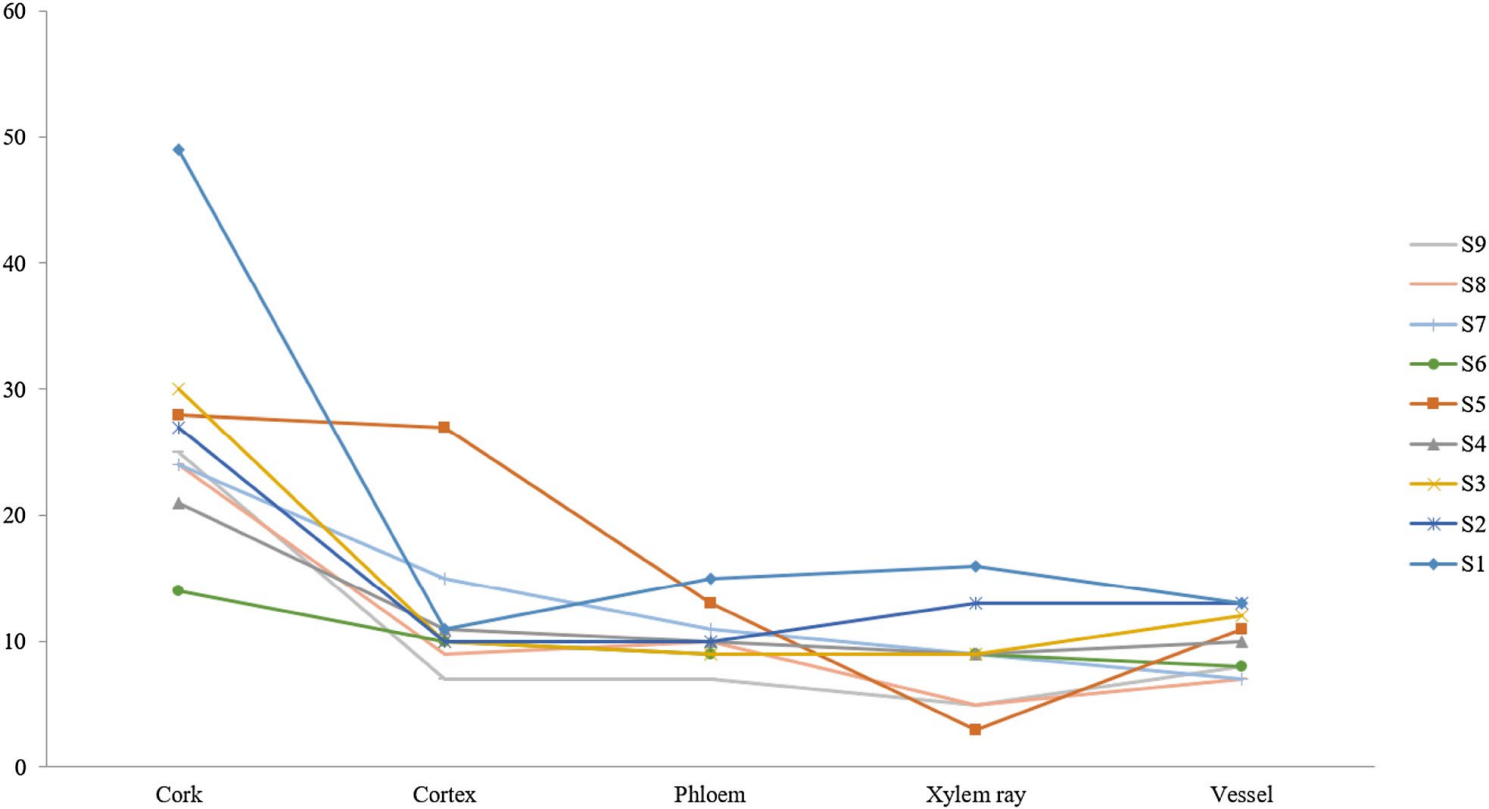
The profile of chemicals in various tissues from S1 to S9

Mapping chemical profiles in micro-dissection tissues was performed by UPLC-QTOF-MS and the representative base peak chromatograms (BPC) showing all the detected peaks of cork and cortex tissues from S1, S2 and S5 were showed in Fig. 3. The BPC chromatograms of others were showed in the Additional file 1. Total 62 chromatographic peaks were detected (Table 3). Peaks of tanshinones could be recognized by their generated molecular ions of [M+Na]^+^ and [M+H]^+^ while peaks of salvianolic acids were easily generated their molecular ions of [M−H]^−^. Peaks 4, 38, 48, 49 and 59 were identified as SaB, DHTan I, Tan I, CTan and Tan II_A_ by their accurate mass and corresponding mass ions as well as comparison of chemical markers, respectively. The molecular ions of SaB (717.1406 [M−H]^−^ m/z), DHTan I (301.0834 [M+Na]^+^ and 279.1015 [M+H]^+^ m/z), Tan I (299.0684 [M+Na]^+^ and 277.0867 [M+H]^+^ m/z), CTan (319.1307 [M+Na]^+^ and 297.1488 [M+H]^+^ m/z) and Tan II_A_ (317.1158 [M+Na]^+^ and 295.1333 [M+H]^+^ m/z) were detected in marker and sample solutions. The molecular ions of others were identified or tentatively characterized by their accurate mass data in comparison with literature reports [14–22]. From the Fig. 4, the number of chemicals in cork was more abundant than those in other tissues from all of samples. In the BPC chromatograms of various tissues, the chemical profiles of 9 samples were dissimilar (Table 4). Peaks 1 and 2, peaks 6 and 7, peak 8 only could be detected in cortex of S5, in cork of S2, in cork of S1, respectively while peak 38 (DHTan I) could be detected in all micro-dissected tissues except for xylem ray of S5. SaB, DHTan I, Tan I, CTan and Tan II_A_ were found as common peaks in cork from all of samples and some of them also could be detected in other tissues. The results demonstrated that SaB, DHTan I, Tan I, CTan and Tan II_A_ were the main components in the tissues of root of *S. miltiorrhiza*. Thus, further quantitative analysis of them in various tissues was also carried out by UPLC-QTOF-MS.

**Table 3:** Characteristics of bioactive components in various tissues

Peak no.^a^	R_t_ (min)	Polarity	Formula	Identification
1	2.63	313.0718 [M−H]^−^	C_17_H_14_O_6_	Salvianolic acid F^b^
2	3.59	535.1818 [M−H]^−^	C_26_H_32_O_12_	(+)1-hydroxypinoresinol-1-O-β-D-glucoside^b^
3	3.96	359.0732 [M−H]^−^	C_18_H_16_O_8_	Rosmarinic acid^b^
4	4.43	717.1406 [M−H]^−^	C_36_H_30_O_16_	Salvianolic acid B^c^
5	4.56	137.0242 [M−H]^−^	C_7_H_6_O_3_	Protocatechualdehyde^b^
6	4.98	193.0479 [M−H]^−^	C_10_H_10_O_4_	Ferulic acid^b^
7	6.04	335.0894 [M+Na]^+^, 313.1071 [M+H]^+^	C_18_H_16_O_5_	Tanshindiol C^b^
8	7.18	297.1118 [M−H]^−^	C_19_H_22_O_3_	Arucadiol^b^
9	7.40	319.0944 [M+Na]^+^, 297.1124 [M+H]^+^	C_18_H_16_O_4_	Danshenxinkun^b^
10	7.63	117.0193 [M−H]^−^	C_4_H_6_O_4_	Succinic acid^b^
11	7.77	357.0588 [M−H]^−^	C_18_H_14_O_8_	Prolithospermic acid^b^
12	7.84	335.1252 [M + Na]^+^, 313.1432 [M+H]^+^	C_19_H_20_O_4_	Miltionone II^b^
13	8.99	317.0786 [M+Na]^+^, 295.0969 [M+H]^+^	C_18_H_14_O_4_	Trijuganone A^b^
14	9.12	319.0944 [M+Na]^+^, 297.1124 [M+H]^+^	C_18_H_16_O_4_	Tanshinone VI^b^
15	9.27	383.9794 [M−H]^−^		Unknown
16	9.98	333.1097 [M+Na]^+^, 311.1279 [M+H]^+^	C_19_H_18_O_4_	Isotanshinone^b^
17	10.07	303.0996 [M+Na]^+^, 281.1162 [M+H]^+^	C_18_H_16_O_3_	Methylene dihydrotanshinone^b^
18	10.21	335.1252 [M+Na]^+^, 313.1434 [M+H]^+^	C_19_H_20_O_4_	Miltionone I^b^
19	10.35	491.1039 [M−H]^−^	C_26_H_20_O_10_	Salvianolic acid C^b^
20	10.41	333.1098 [M+Na]^+^, 311.1282 [M+H]^+^	C_19_H_18_O_4_	Tanshinone II_B_^b^
21	10.64	333.1100 [M+Na]^+^, 311.1282 [M+H]^+^	C_19_H_18_O_4_	3α-hydroxytanshinone II_A_/3β-hydroxytanshinone II_A_^b^
22	10.87	333.1099 [M+Na]^+^, 311.1283 [M+H]^+^	C_19_H_18_O_4_	3α-hydroxytanshinone II_A_/3β-hydroxytanshinone II_A_^b^
23	10.98	327.0872 [M−H]^−^	C_18_H_16_O_6_	Methylsalvianolate F^b^
24	11.38	363.1202 [M+Na]^+^, 341.1380 [M+H]^+^	C_20_H_20_O_5_	Cryptomethyltanshinoate^b^
25	11.57	295.0958 [M−H]^−^	C_18_H_16_O_4_	Tanshinol B^b^
26	11.75	325.1079 [M−H]^−^	C_14_H_14_O_9_	Monocaffeoyltartaric acid^b^
27	12.01	285.1853 [M−H]^−^	C_20_H_30_O	Ferruginol^b^
28	12.02	309.1125 [M+Na]^+^, 287.1642 [M+H]^+^	C_18_H_22_O_3_	Epicryptoacetalide/Cryptoacetalide^b^
29	12.18	487.3401 [M−H]^−^		Unknown
30	12.24	309.1125 [M+Na]^+^, 287.2002 [M+H]^+^	C_18_H_22_O_3_	Epicryptoacetalide/Cryptoacetalide^b^
31	12.35	313.1438 [M−H]^−^	C_19_H_22_O_4_	Tanshinone V^b^
32	12.38	301.0838 [M+Na]^+^, 279.1016 [M+H]^+^	C_18_H_14_O_3_	Methylenetanshinquinone^b^
33	12.46	485.3274 [M−H]^−^		Unknown
34	12.57	537.1038 [M−H]^−^	C_27_H_22_O_12_	Lithospermic acid^b^
35	12.82	293.0819 [M−H]^−^	C_18_H_14_O_4_	3-hydroxymethylenetanshinone^b^
36	12.88	321.1646 [M+Na]^+^, 299.1642 [M+H]^+^	C_19_H_22_O_3_	Miltiodiol^b^
37	12.96	555.3268 [M−H]^−^		Unknown
38	13.00	301.0834 [M+Na]^+^, 279.1015 [M+H]^+^	C_18_H_14_O_3_	Dihydrotanshinone I^c^
39	13.11	301.0834 [M+Na]^+^, 279.1015 [M+H]^+^	C_18_H_14_O_3_	1,2-dihydrotanshinone I^b^
40	13.16	329.1750 [M−H]^−^	C_20_H_26_O_4_	Salviol^b^
41	13.41	319.1306 [M+Na]^+^, 297.1491 [M+H]^+^	C_19_H_20_O_3_	Isocryptotanshinone^b^
42	13.84	303.0998 [M+Na]^+^, 281.1173 [M+H]^+^	C_18_H_16_O_3_	Danshenxinkun B^b^
43	14.25	361.1045 [M+Na]^+^, 339.1230 [M+H]^+^	C_20_H_18_O_5_	Methyl tanshinoate^b^
44	14.32	357.0616 [M−H]^−^	C_18_H_14_O_8_	Prolithospermic acid^b^
45	14.62	333.1089 [M+Na]^+^, 301.1800 [M+H]^+^	C_19_H_24_O_3_	Miltipolone^b^
46	14.75	265.1470 [M−H]^−^	C_18_H_18_O_2_	Methylenemiltirone^b^
47	15.45	315.0846 [M−H]^−^	C_17_H_16_O_6_	5,3′-dihydroxy-7,4′-dimethoxyflavanone^b^
48	15.97	299.0684 [M+Na]^+^, 277.0867 [M+H]^+^	C_18_H_12_O_3_	Tanshinone I^c^
49	16.00	319.1307 [M+Na]^+^, 297.1488 [M+H]^+^	C_19_H_20_O_3_	Cryptotanshinone^c^
50	16.15	315.1949 [M−H]^−^	C_20_H_28_O_3_	1-​phenanthrenecarboxyl​ic acid^b^
51	16.35	297.1830 [M−H]^−^	C_20_H_26_O_2_	5-​dehydrosugiol^b^
52	16.64	299.2018 [M−H]^−^	C_20_H_28_O_2_	Sugiol^b^
53	16.79	299.0684 [M+Na]^+^, 277.0867 [M+H]^+^	C_18_H_12_O_3_	Isotanshinone I^b^
54	17.25	301.0834 [M+Na]^+^, 279.1015 [M + H]^+^	C_18_H_14_O_3_	Dihydroisotanshinone I^b^
55	17.75	315.1001 [M+Na]^+^, 293.1179 [M+H]^+^	C_19_H_16_O_3_	1,2 -didehydrotanshinone II_A_^b^
56	18.18	289.1204 [M+Na]^+^, 267.1386 [M+H]^+^	C_17_H_14_O_3_	Dihydrotanshinlactone^b^
57	18.87	303.1306 [M+Na]^+^, 281.1539 [M+H]^+^	C_19_H_20_O_2_	Δ^1^ -dehydromiltirone^b^
58	19.13	325.1824 [M−H]^−^	C_21_H_26_O_3_	2-(7-Dihydroxyl)-benzofuranyl-,ferulic acid^b^
59	19.30	317.1158 [M+Na]^+^, 295.1333 [M+H]^+^	C_19_H_18_O_3_	Tanshinone II _A_^c^
60	20.01	317.1151 [M+Na]^+^, 295.1332 [M+H]^+^	C_19_H_18_O_3_	Isotanshinone II _A_^b^
61	20.39	305.1515 [M+Na]^+^, 283.1700 [M+H]^+^	C_19_H_22_O_2_	Miltirone^b^
62	21.31	683.4317 [M−H]^−^	C_44_H_60_O_6_	3,4-Dihydroxy-(1α,3α,4α,5β)-1-carboxy-4-hydroxy-1,3,5-cyclohexanetriyl ester-benzenepropanoic^b^

*R*_*t*_ *r*etention time

^a^The peak numbers referred to Fig. 3

^b^Identified by previously reported from *Salvia* species

^c^Identified by chemical markers

**Table 4:** The distribution of bioactive components in various tissues from different samples

Sample no.	Herbal tissues/peak No.^a^				
	Cork	Cortex	Phloem	Xylem ray	Vessel
S1	3, 4, 8, 9, 12–14, 16–22, 24–39, 41–57, 59–61	9, 17, 32, 36, 38, 39, 41, 46, 48, 49, 53	9, 17, 24, 32, 36, 38, 39, 41, 42, 46, 48, 49, 53, 54, 59	9, 17, 20, 32, 36, 38, 39, 41–43, 48, 49, 53, 54, 59, 61	10, 14, 32, 36, 38, 39, 41, 46, 48, 49, 53, 54, 59
S2	4, 6, 7, 9, 17, 20, 24, 28, 31, 32, 36, 38, 39, 41–43, 45, 46, 48, 49, 52–56, 59, 61	9, 32, 36, 38, 39, 41, 48 49, 53, 54	9, 32, 36, 38, 39, 41, 48, 49, 53, 54	9, 17, 23, 28, 32, 36, 38, 39, 41, 48, 49, 53, 54	9, 17, 24, 28, 32, 36, 38, 39, 41, 48, 49, 53, 54
S3	4, 9, 12–14, 16–18, 20–22, 24, 28, 30, 32, 36, 38, 39, 41–43, 45, 48, 49, 51–55, 59, 61, 62	9, 17, 22, 32, 38, 39, 41 48, 49, 53, 54	9, 32, 38, 39, 41, 48, 49, 53, 54	9, 32, 38, 39, 41, 48, 49, 53, 54	9, 17, 24, 28, 32, 38, 39, 41, 48, 49, 53, 54
S4	4, 5, 9, 14, 20, 30, 32, 36, 38, 39, 41–43, 45, 48, 49, 53–55, 59, 61	9, 14, 20, 32, 38, 39, 41 48, 49, 53, 54	9, 17, 32, 38, 39, 41, 48, 49, 53, 54	9, 32, 38, 39, 41, 48, 49, 53, 54	9, 32, 38, 39, 41, 48, 49, 53,
S5	4, 14, 16, 20, 23, 30, 32, 36, 38, 39, 41–46, 48, 49, 52, 54, 55, 59, 61, 62	1–5, 9–11, 14, 15, 17, 22–24, 28, 32, 36, 38, 39, 41, 48, 49, 53, 58	9, 17, 22, 24, 28, 32, 38, 39, 41, 46, 48, 49, 53	16, 24, 39	9, 10, 32, 38, 39, 41, 48, 49, 53, 54, 59
S6	4, 23, 30, 32, 38, 39, 41, 42, 46, 48, 49, 54, 59, 61	9, 23, 24, 30, 32, 36, 38, 39, 41, 49	23, 24, 30, 32, 38, 39, 41, 46, 48	9, 23, 24, 30, 32, 38, 39, 41, 46	23, 30, 32, 36, 38, 39, 41, 46
S7	4, 12, 14, 16, 18, 20, 23, 24, 30, 32, 36, 38, 41–43, 45, 46, 48–50, 52, 54, 59, 61	9, 16, 23, 24, 30, 32, 37–39, 41, 42, 45, 48, 49, 53	9, 23, 24, 30, 32, 38, 39, 41, 46, 49, 63	9, 23, 24, 30, 32, 38, 39, 41, 49	23, 30, 32, 38, 39, 41, 49
S8	4, 12, 14, 16, 18, 20, 23, 24, 30, 32, 36, 38, 41–43, 45, 48–50, 52, 54, 59, 61	4, 23, 24, 30, 32, 38, 39, 41, 49	23, 24, 30, 32, 36, 38, 39, 41, 46, 49	23, 30, 32, 38, 39	4, 23, 30, 32, 38, 39, 49
S9	4, 12, 17, 22–33, 35, 37–42, 48, 49, 59	23, 30, 32, 36, 38, 39, 41	23, 30, 32, 36, 38, 39, 41	23, 30, 32, 38, 39	23, 30, 32, 36, 38, 39, 41

^a^The peak numbers referred to Table 3 and Fig. 3

### Quantitative analysis of tanshinones and salvianolic acids in various tissues

Linear regression analysis in statistics including calibration curve and correlation coefficients of determination (R2), limits of detection (LOD, S/N > 3) and limits of quantification (LOQ, S/N > 10) were investigated under the above conditions for the quantitative analysis. The peak areas as the dependent variable (y axis) and the concentration as the independent variable (x axis, ng/mL) was used to generate the calibration curves of each reference, All of the R^2^ value were over 0.9996 (n = 9). The LOD is 44.31, 3.88, 7.73, 3.87 and 8.03 ng/mL to Sa B, DHTan I, Tan I, CTan and Tan IIA and the LOQ is 75.00, 12.90, 12.42, 12.89 and 26.74 ng/mL to Sa B, DHTan I, Tan I, CTan and Tan IIA, respectively (Table 5).

**Table 5:** Methodological validation data of chemical markers

Chemical markers	Calibration curve	R^2^	LOD (ng/mL)	LOQ (ng/mL)
Sa B	Y = 34.82X−5199.5	0.9997	44.31	75.00
DHTan I	Y = 903.46X+2021.7	0.9996	3.88	12.90
Tan I	Y = 245.31X+1718.4	0.9997	3.73	12.42
CTan	Y = 1410.80X+1063.5	1.0000	3.87	12.89
Tan II _A_	Y = 1531.80X+12447	0.9998	8.03	26.74

**Fig. 5:**
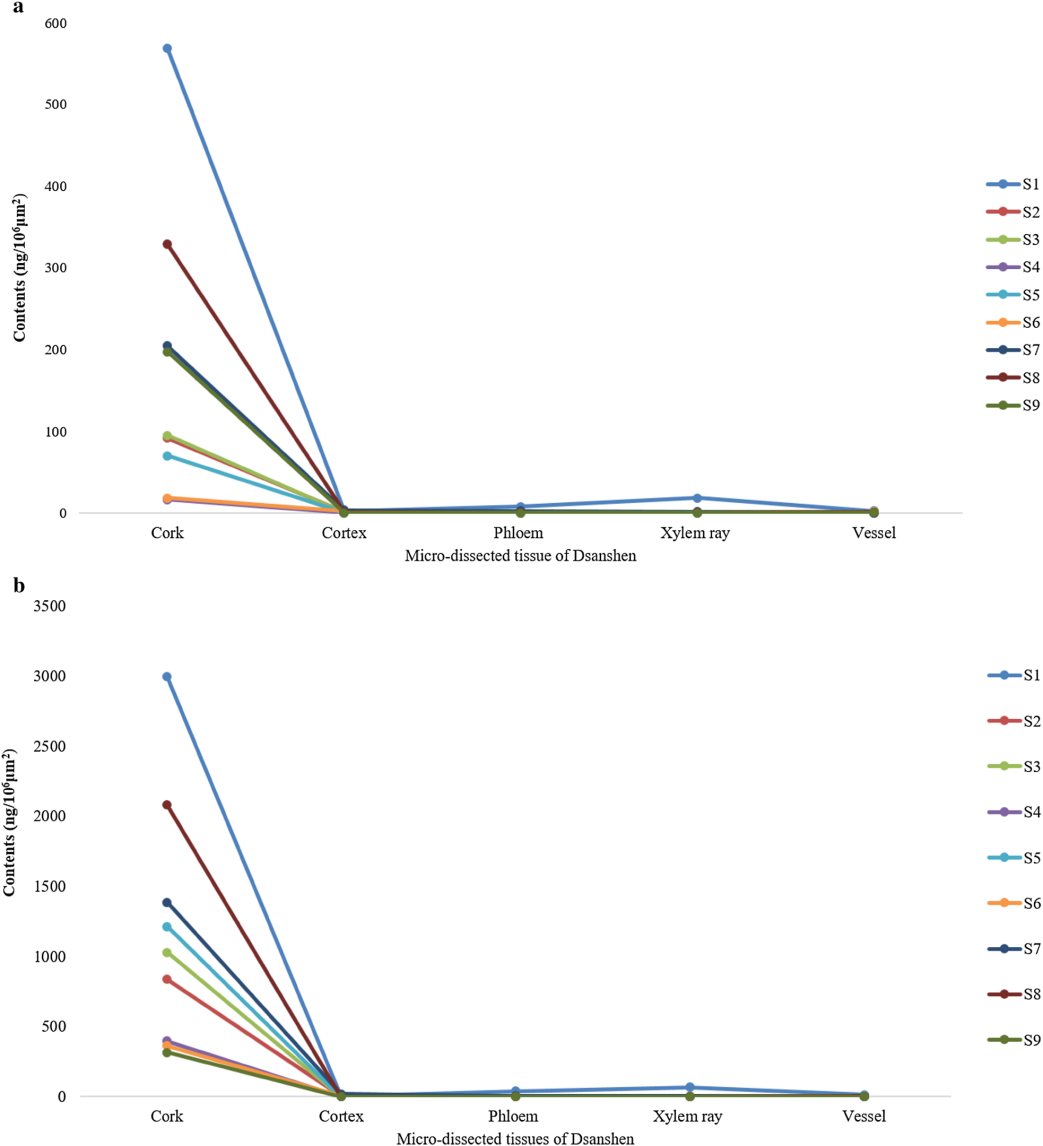
Methodological validation data of chemical markers

**Fig. 6:**
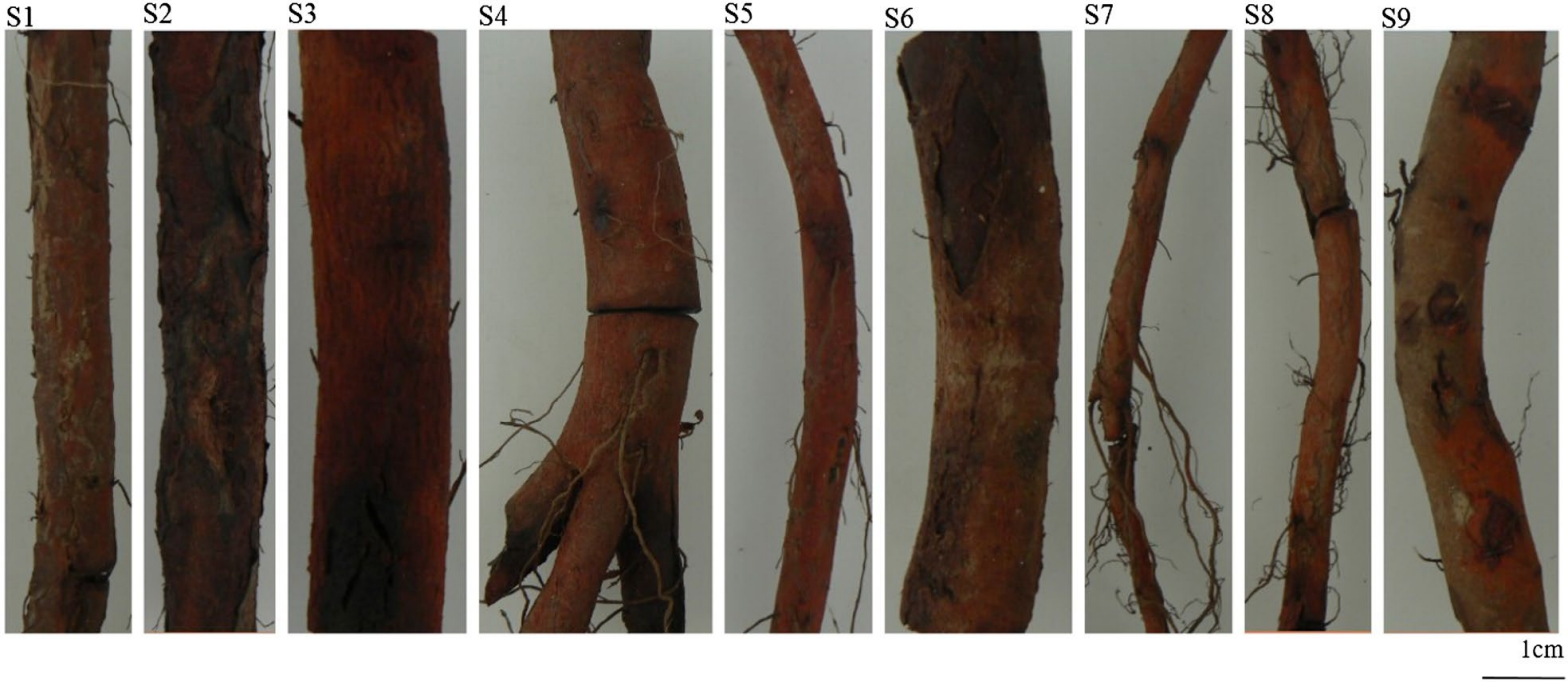
The appearance of 9 research samples (S1–S9, from *left* to *right*)

**Fig. 7:**
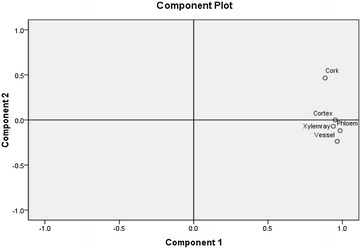
A loading plot obtained from principal component analysis of the contents of major tanshinones contained in different tissues from all of samples

The results (Fig. 5) demonstrated that the amounts of major tanshinones and Sa B in various tissues were different and it could be seen that the contents of Sa B (Fig. 5a) and major tanshinones (Fig. 5b, calculated by DHTan I, Tan I, CTan and Tan IIA) in cork were much higher than those in other herbal tissues as well. In addition, Sa B could also be quantified in cortex of samples 5 and 8. It suggested that the growing area and/or harvest season could influence tissue-specific chemical profiles, especially affect the amounts of major tanshinones. In detail, the total contents of major tanshinones in cork from different samples were distinct. The amounts of major tanshinones in S1 were highest and those in S9 were much lower than other samples. For Tan IIA, the same phenomenon was also found. Even from the same growing area, it was also different. S6, S7 and S8 were from Beijing growing area, the size of S8 was smaller than S6 but it contained higher amounts of major tanshinones, reaching around sixfold to S6 while the size of S8 and S7 was similar but it contained higher contents than those of S6 (Fig. 6; Table 1). This may be connected to the cultivation technologies. Distinctly, even though S1 was not the biggest size of main root in research samples, the total contents of major tanshinones were the highest among all of samples. Modern studies on quality evaluation have demonstrated that the roots of *S. miltiorrhiza* from Zhong Jiang county located in Sichuan province of China have the best pharmaceutical quality and this production district has been regarded as one of geo-authentic habitats of *S. miltiorrhiza* [3]. Principal component analysis was used to compare amounts of major tanshinones in different tissues from all herbal samples in order to further verify experimental results. The loading plot (Fig. 7) showed that the cork and other tissues were obviously separated by the two most important principal components. Moreover, only cork showed brown–red or dark brown–red whether it was observed in bright filed or in fluorescence mode and the total contents of major tanshinones in cork were much higher than those of other tissues among all of samples. Thus, tanshinones may be responsible for the unequal fluorescence characteristics between cork and other tissues.

## Conclusions

In conclusion, different tissues from the same sample and different samples have various chemical profiles. The total contents of salvianolic acid B and major tanshinones varied in samples from the same or different growing areas and different harvest seasons.

As mentioned before, traditional experience on quality evaluation of Danshen considers that the main root with bigger size and deeper brown–red has better pharmaceutical quality [23]. Now, the present study has revealed that its major active components such as tanshinones and salvia acids are mainly accumulated in cork tissue and higher amounts of tanshinones in cork would exhibit deeper brown–red. Thus, Danshen with thinner main root, more lateral roots and deeper brown–red of outer bark would contain higher tanshinone components. The results support one of the criteria of traditional pharmaceutical quality evaluation of Danshen that samples with deeper brown red of outer bark have better quality. However, it is contradicted with another criterion which samples with bigger size of main root have better quality. It is to say that bigger main root of this herbal medicine cannot ensure better pharmaceutical quality. Also, the factors of influencing the pharmaceutical quality involve production district, harvest season and cultivation technologies. For the quality evaluation by morphological features with size of main root and color of outer bark should be restricted to the samples from the same growing area with the same harvest season and cultivation technique. Therefore, comprehensive quality evaluation system of Danshen including morphological features as well as qualitative and quantitative analysis of chemicals should be established.

## Additional file

10.1186/s13065-016-0187-7 Supplementary data involving the BPC chromatograms of various micro-dissected tissues from samples 1–9 were provided.
