# Supplementary material for: Tissues-based chemical profiling and semi-quantitative analysis of bioactive components in the root of Salvia miltiorrhiza Bunge by using laser microdissection system combined with UPLC-q-TOF-MS
Source: Chem Cent J. 2016 Jul 13;10:42. doi: 10.1186/s13065-016-0187-7 (PMC4944434; doi:10.1186/s13065-016-0187-7)
Supplement: Supplementary file 1 — 10.1186/s13065-016-0187-7 Supplementary data involving the BPC chromatograms of various micro-dissected tissues from samples 1–9 were provided. [file 13065_2016_187_MOESM1_ESM.pptx]

## Slide 1
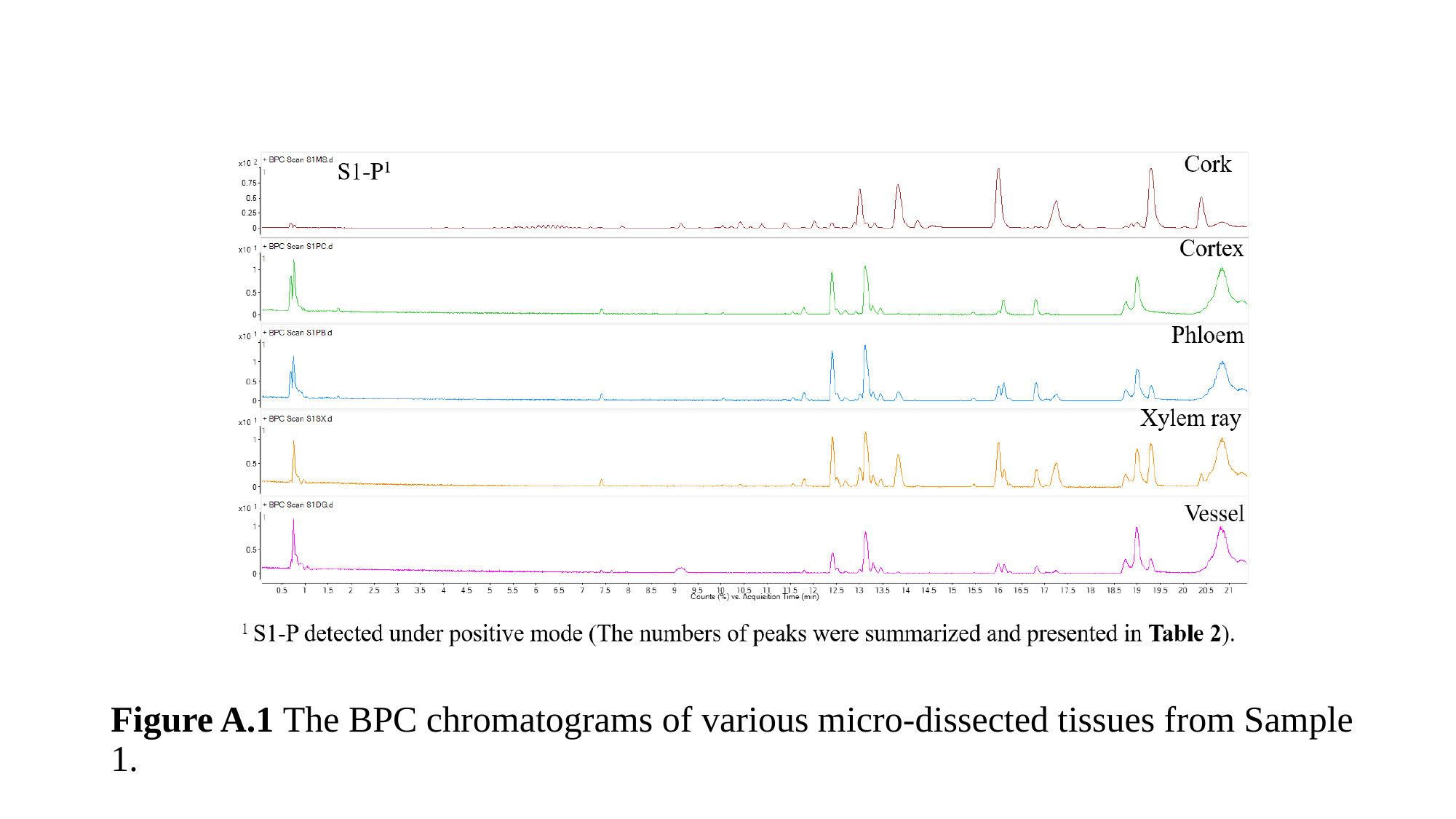

# Figure A.1 The BPC chromatograms of various micro-dissected tissues from Sample 1.

## Slide 2
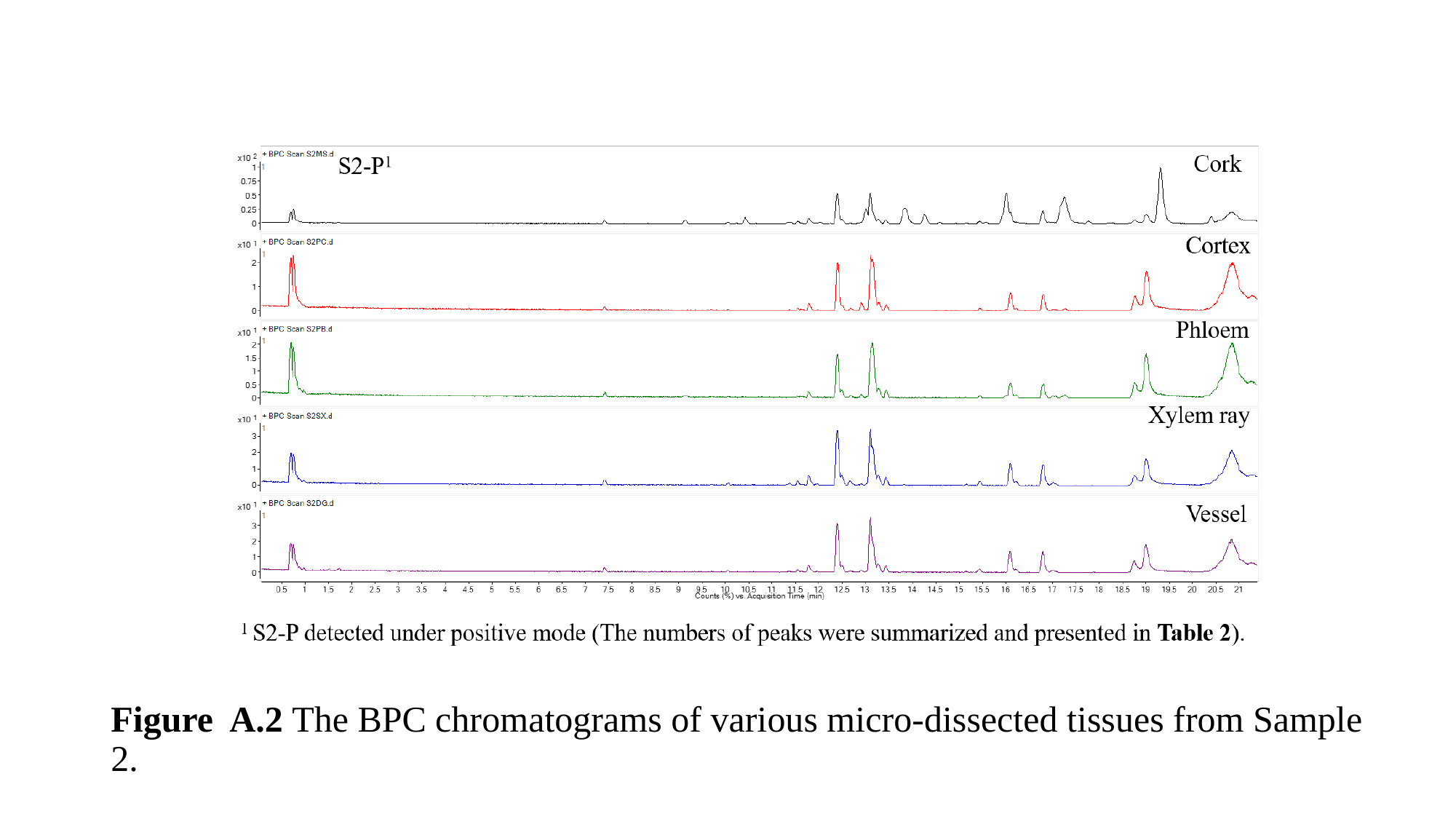

# Figure A.2 The BPC chromatograms of various micro-dissected tissues from Sample 2.

## Slide 3
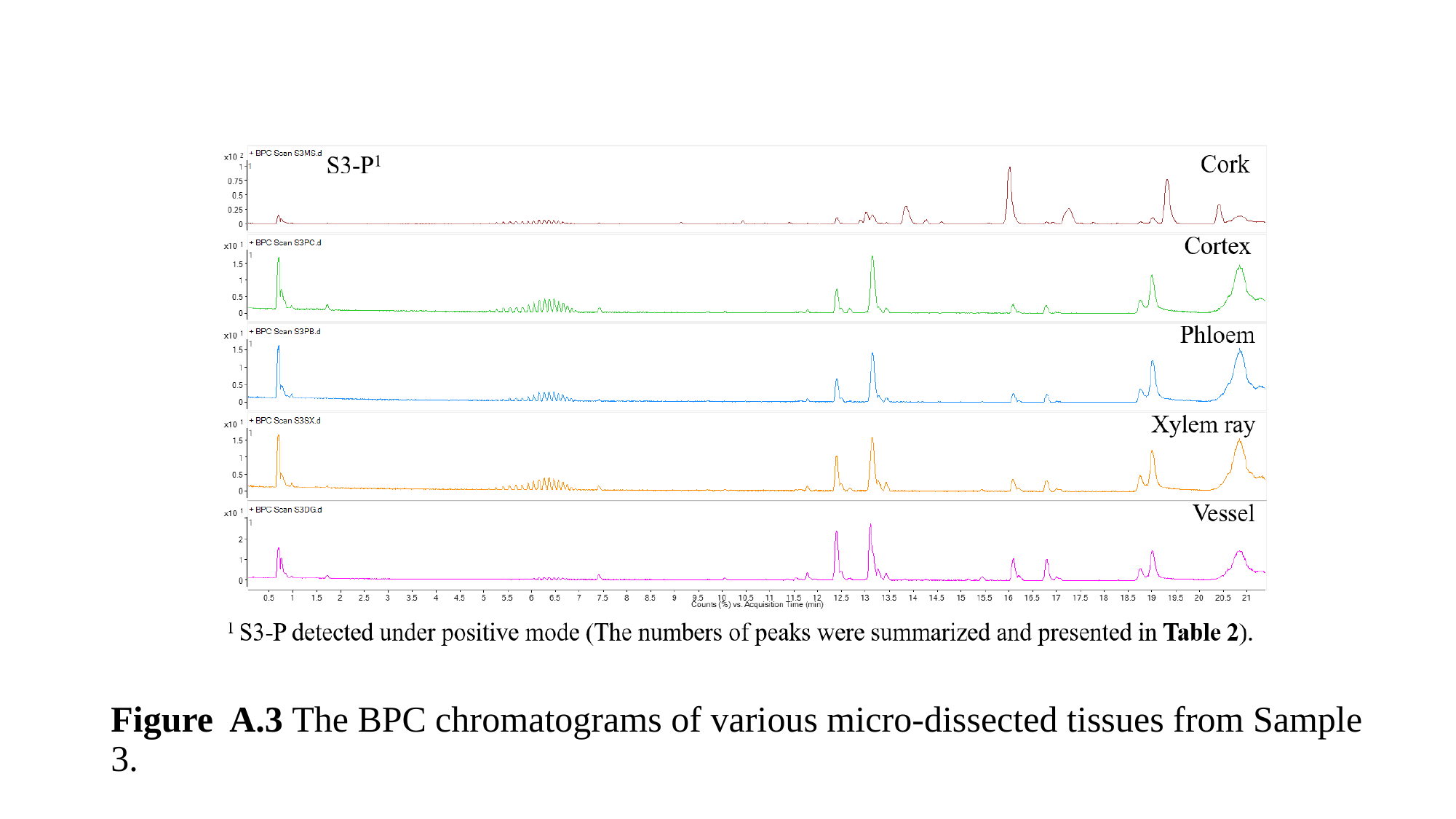

# Figure A.3 The BPC chromatograms of various micro-dissected tissues from Sample 3.

## Slide 4
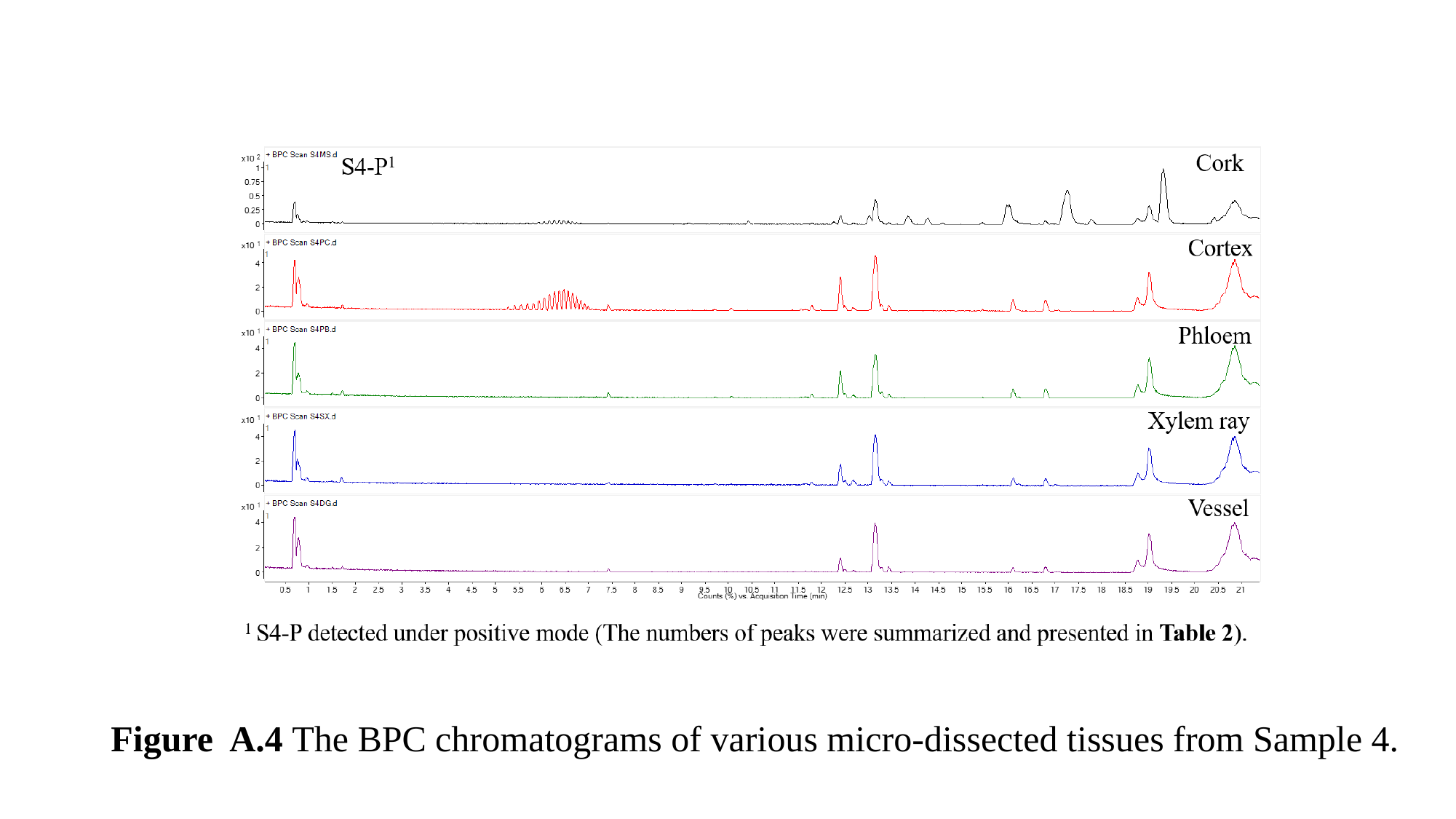

# Figure A.4 The BPC chromatograms of various micro-dissected tissues from Sample 4.

## Slide 5
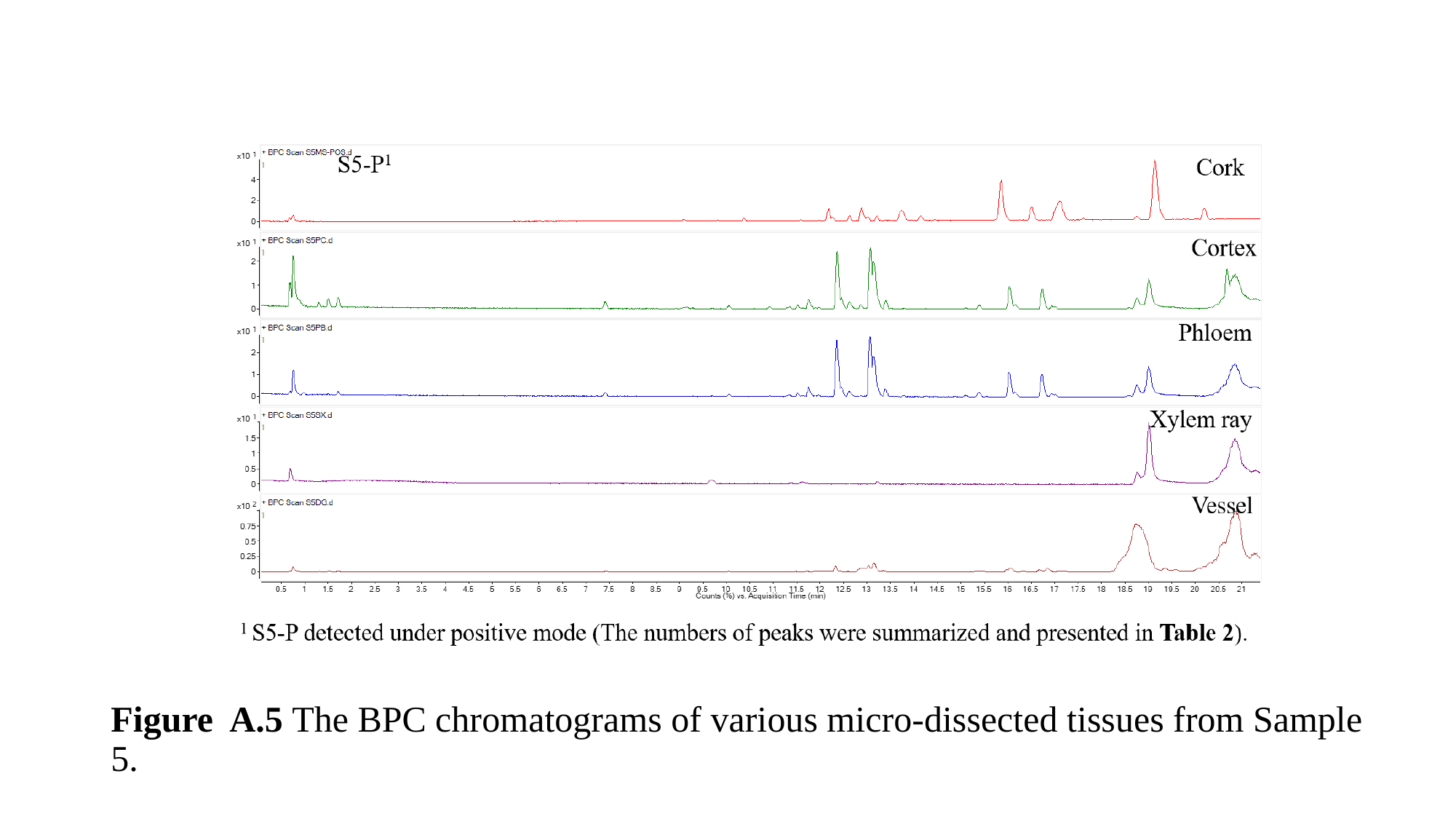

# Figure A.5 The BPC chromatograms of various micro-dissected tissues from Sample 5.

## Slide 6
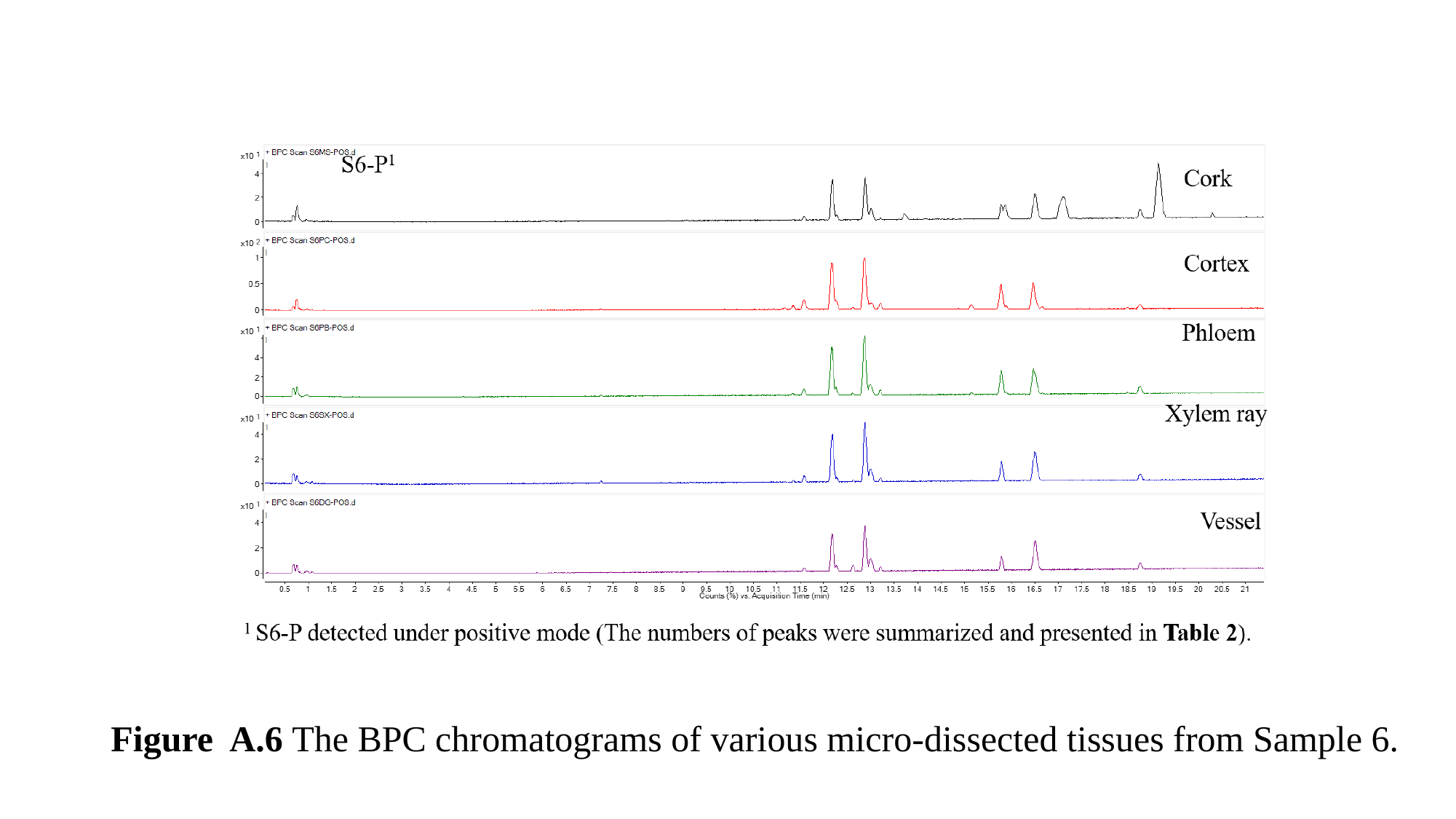

# Figure A.6 The BPC chromatograms of various micro-dissected tissues from Sample 6.

## Slide 7
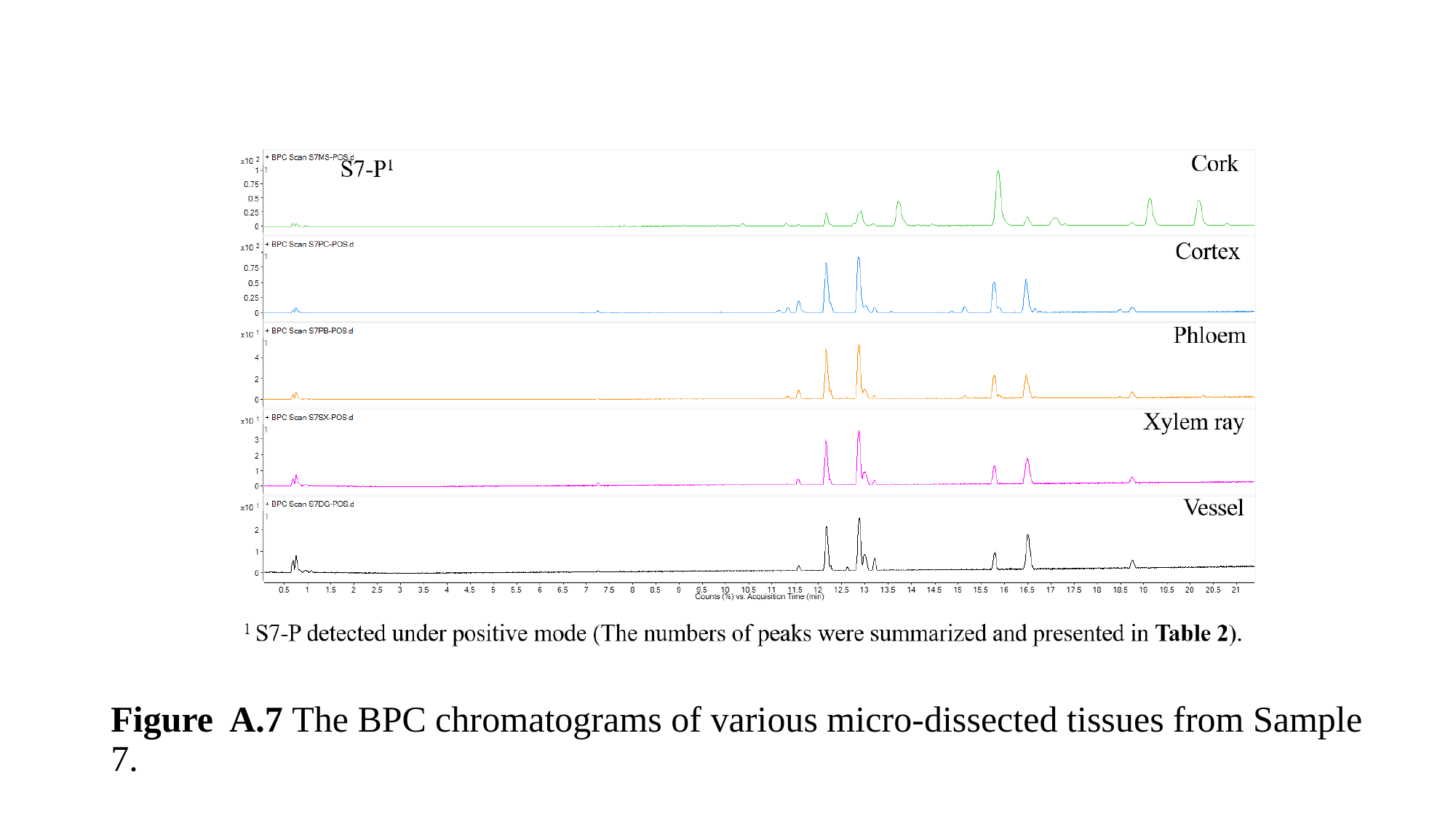

# Figure A.7 The BPC chromatograms of various micro-dissected tissues from Sample 7.

## Slide 8
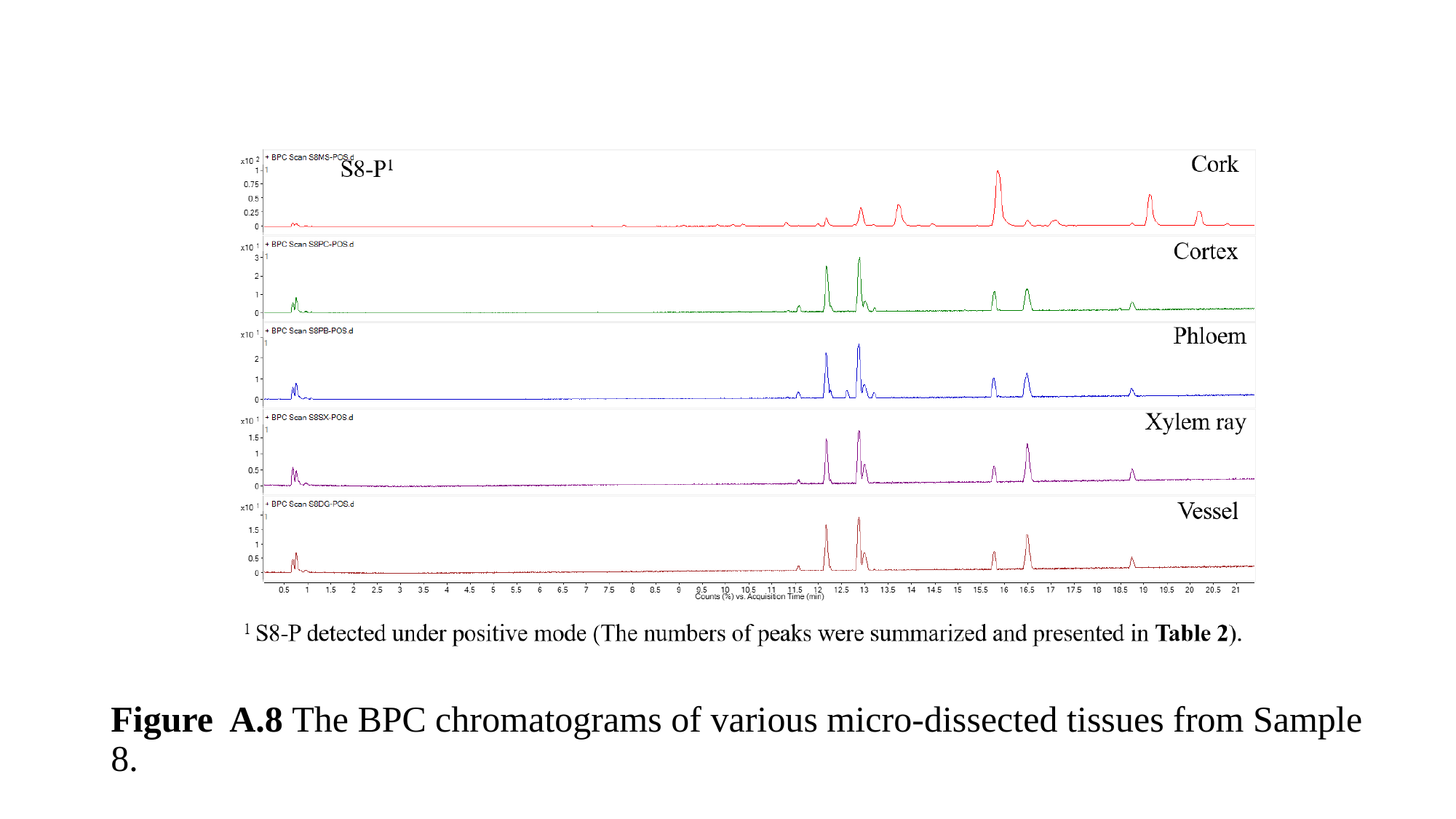

# Figure A.8 The BPC chromatograms of various micro-dissected tissues from Sample 8.

## Slide 9
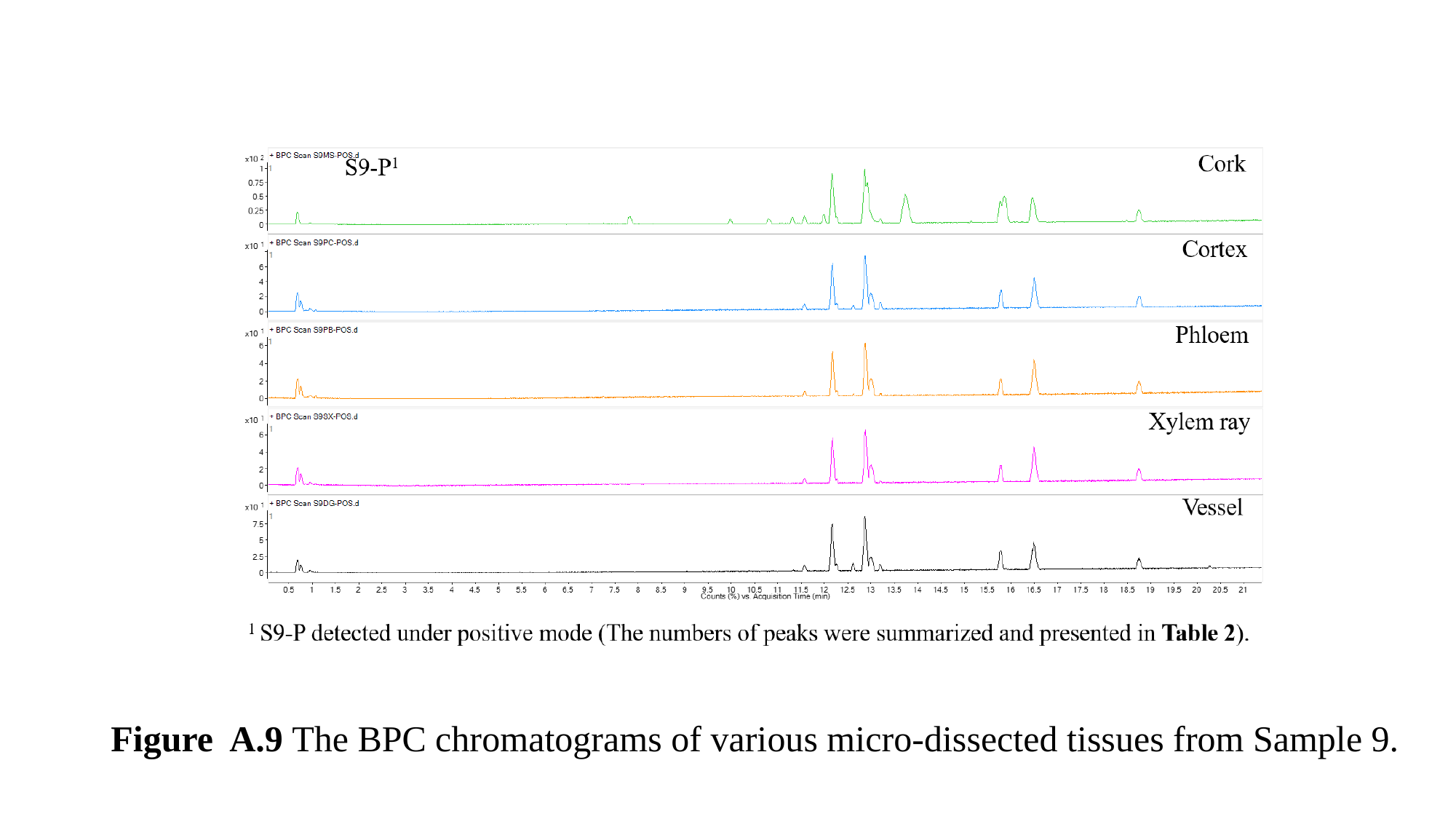

# Figure A.9 The BPC chromatograms of various micro-dissected tissues from Sample 9.

## Slide 10
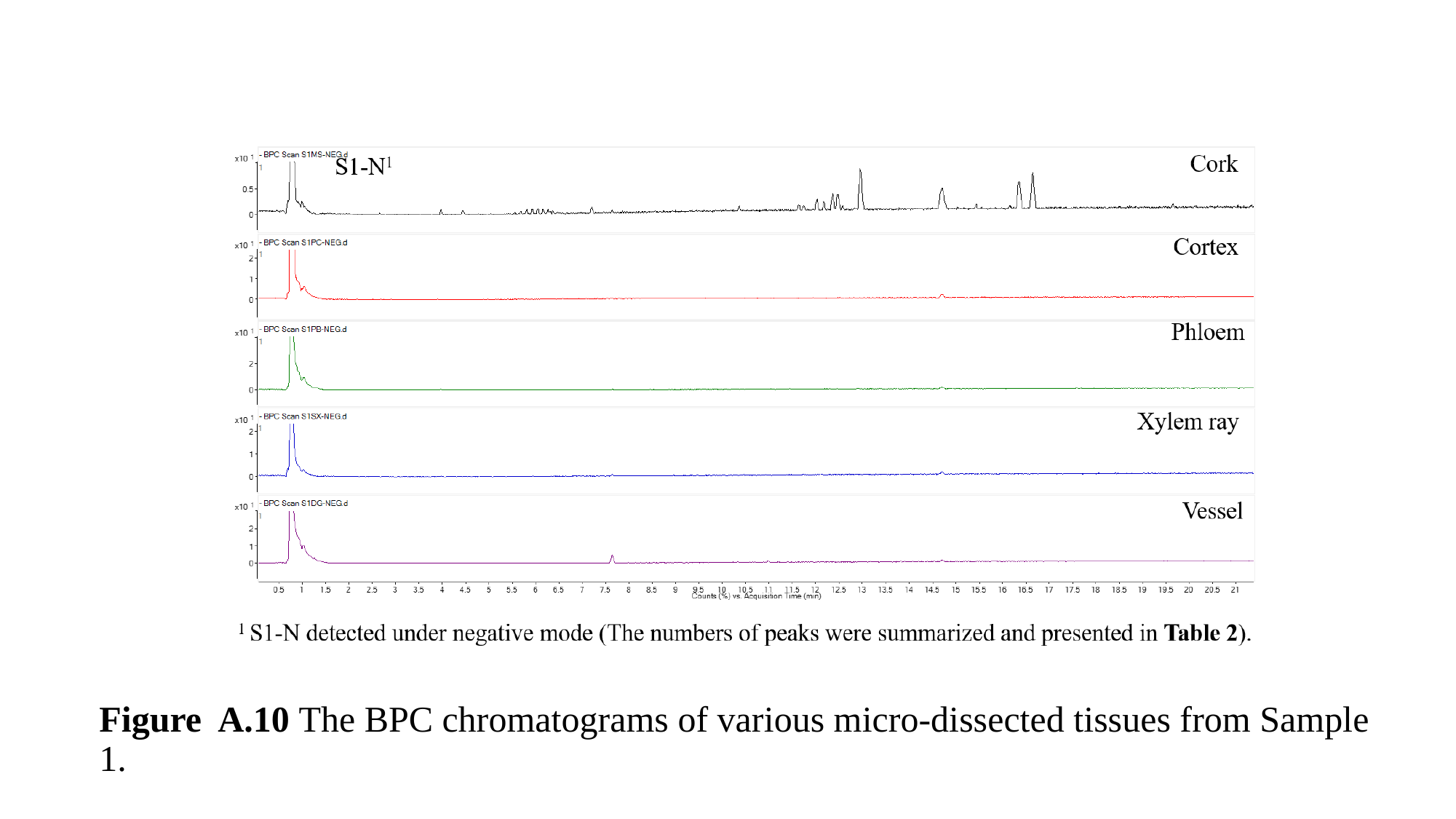

# Figure A.10 The BPC chromatograms of various micro-dissected tissues from Sample 1.

## Slide 11
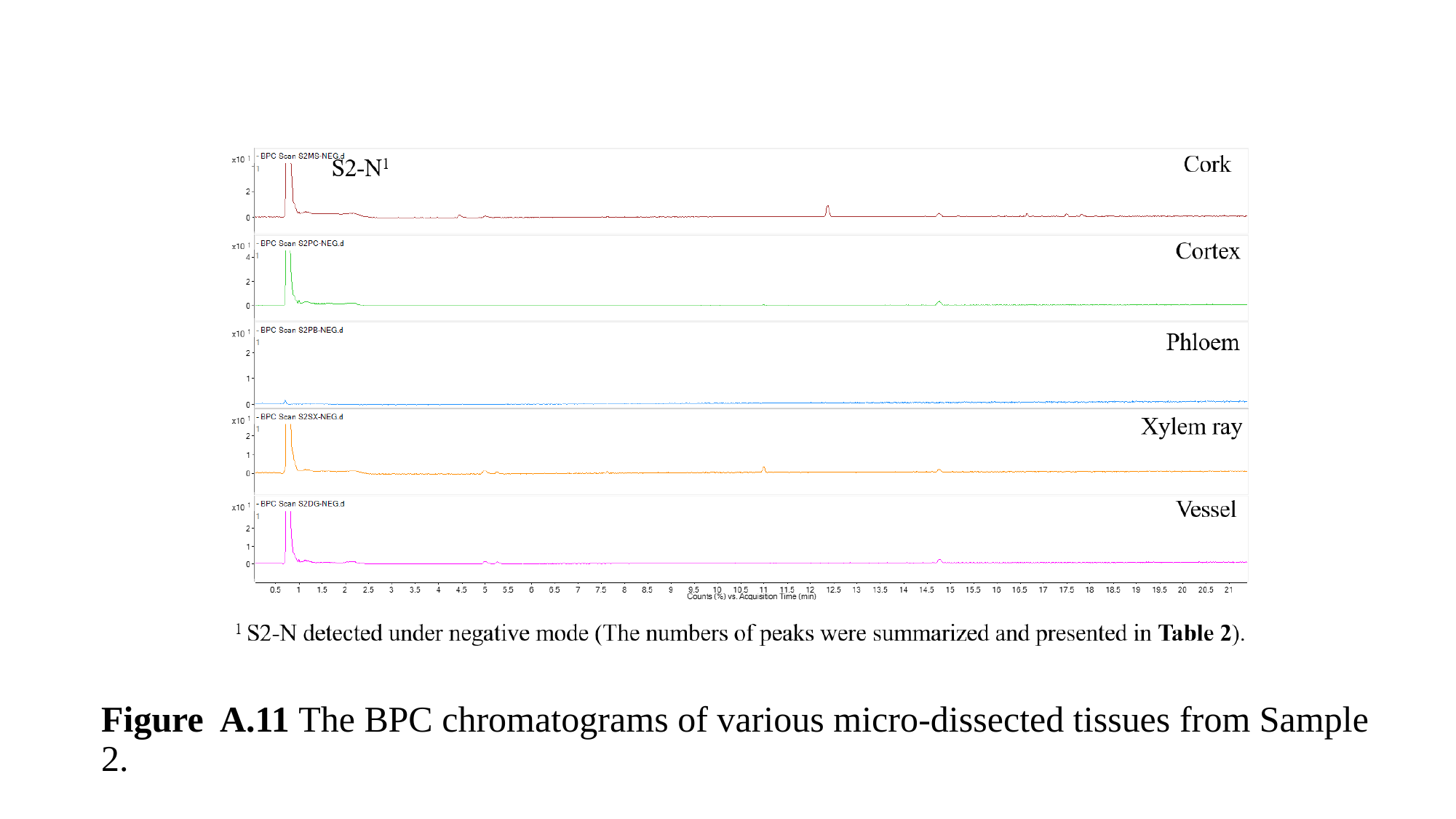

# Figure A.11 The BPC chromatograms of various micro-dissected tissues from Sample 2.

## Slide 12
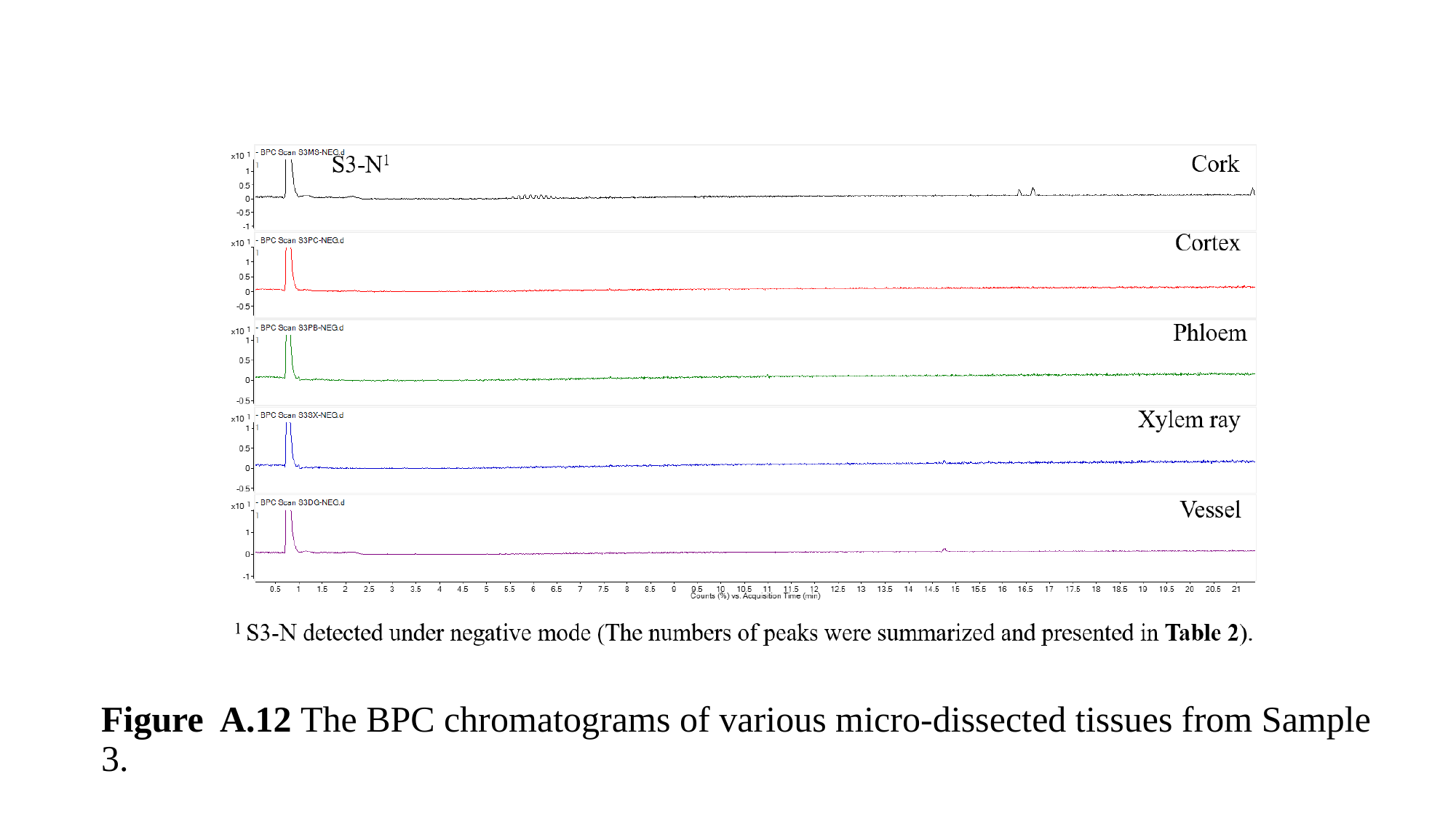

# Figure A.12 The BPC chromatograms of various micro-dissected tissues from Sample 3.

## Slide 13
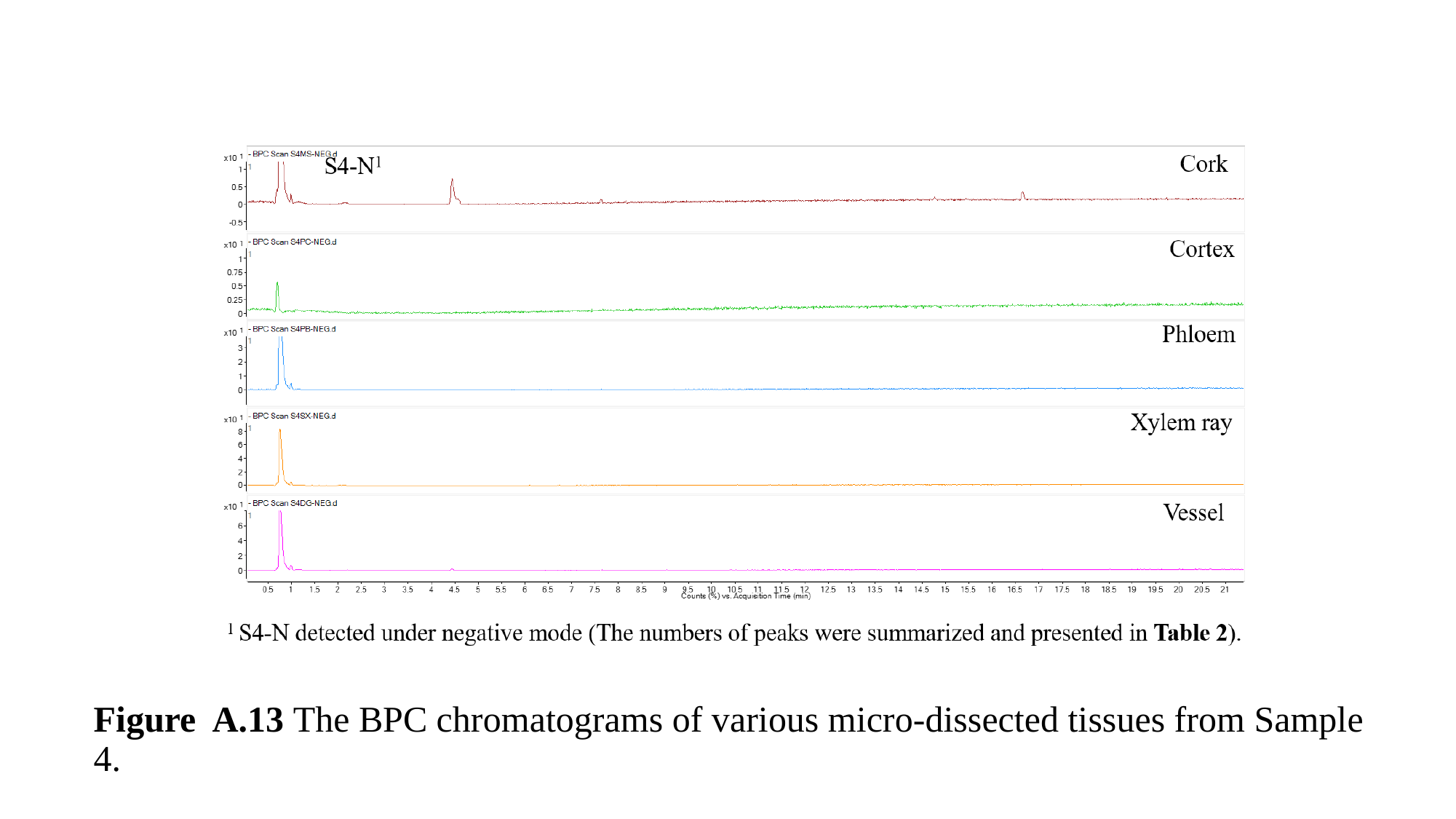

# Figure A.13 The BPC chromatograms of various micro-dissected tissues from Sample 4.

## Slide 14
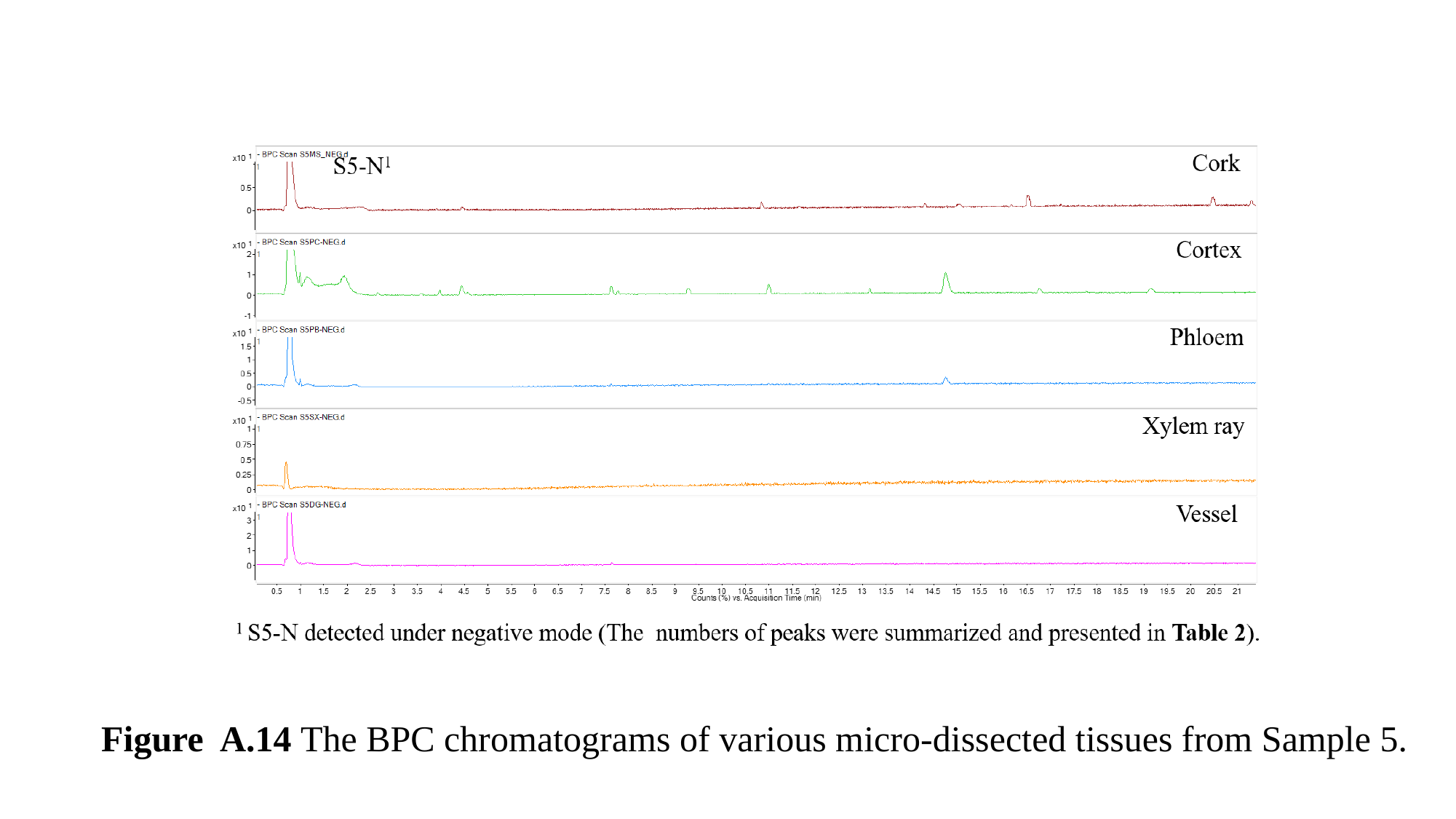

# Figure A.14 The BPC chromatograms of various micro-dissected tissues from Sample 5.

## Slide 15
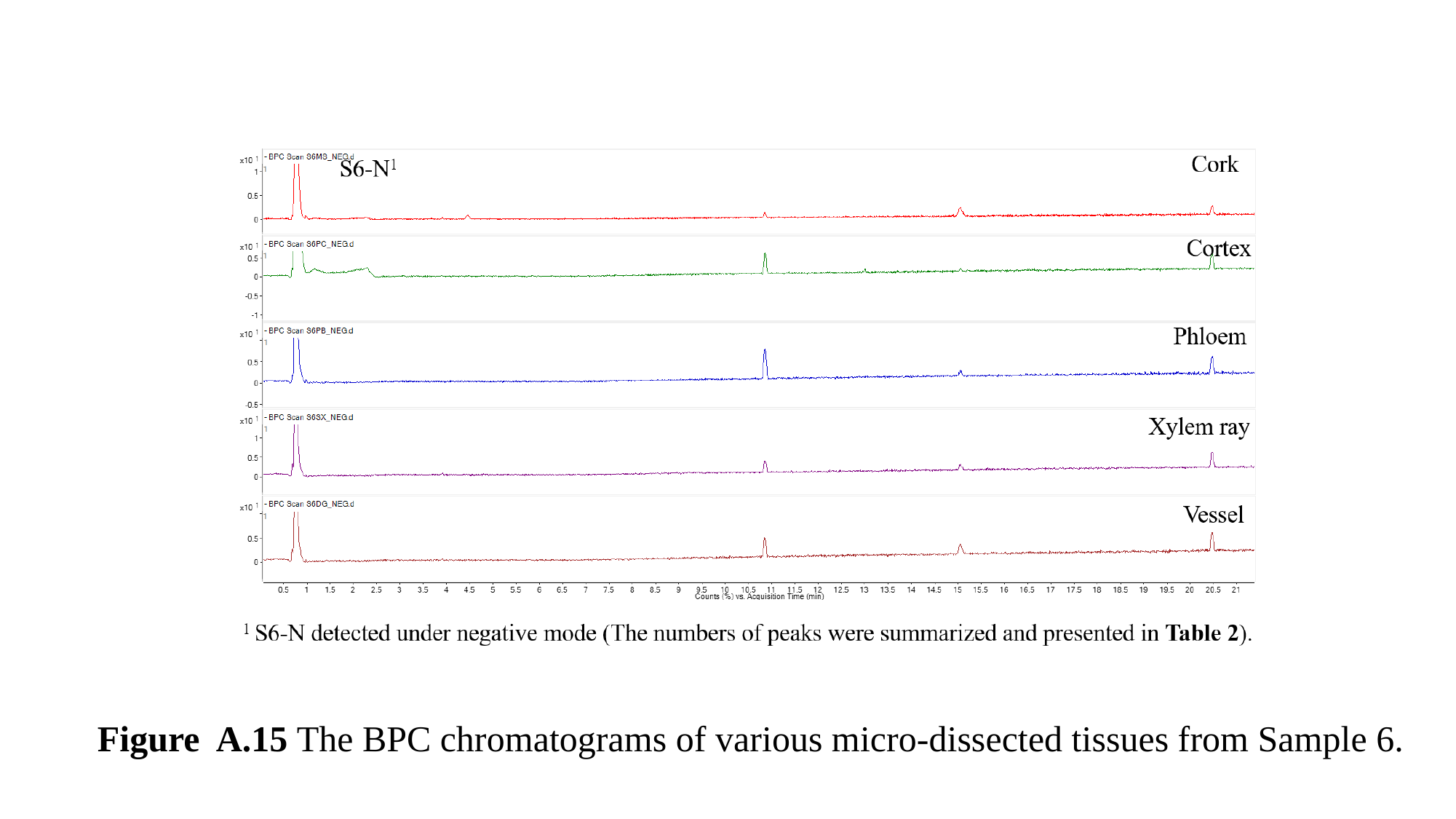

# Figure A.15 The BPC chromatograms of various micro-dissected tissues from Sample 6.

## Slide 16
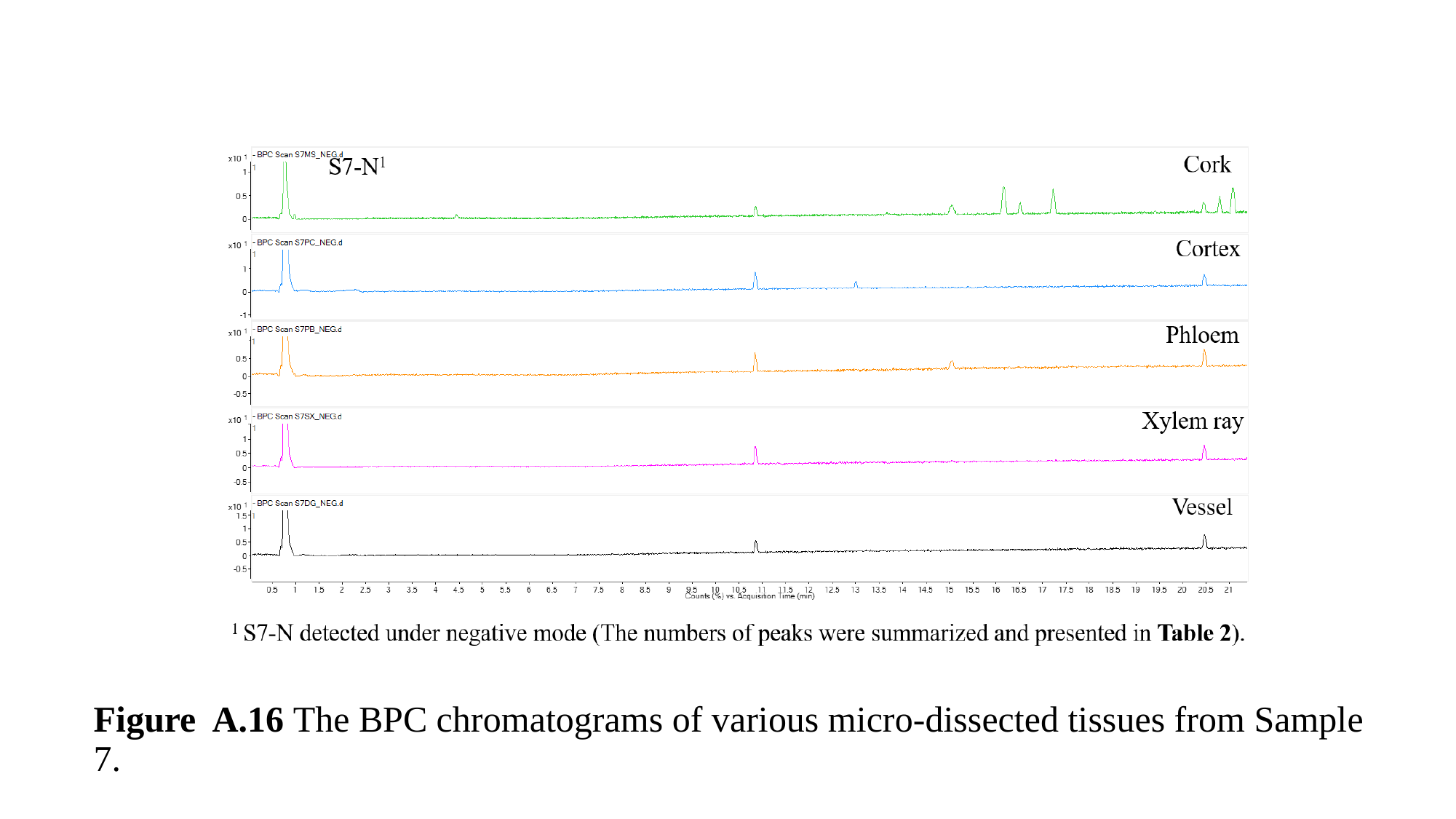

# Figure A.16 The BPC chromatograms of various micro-dissected tissues from Sample 7.

## Slide 17
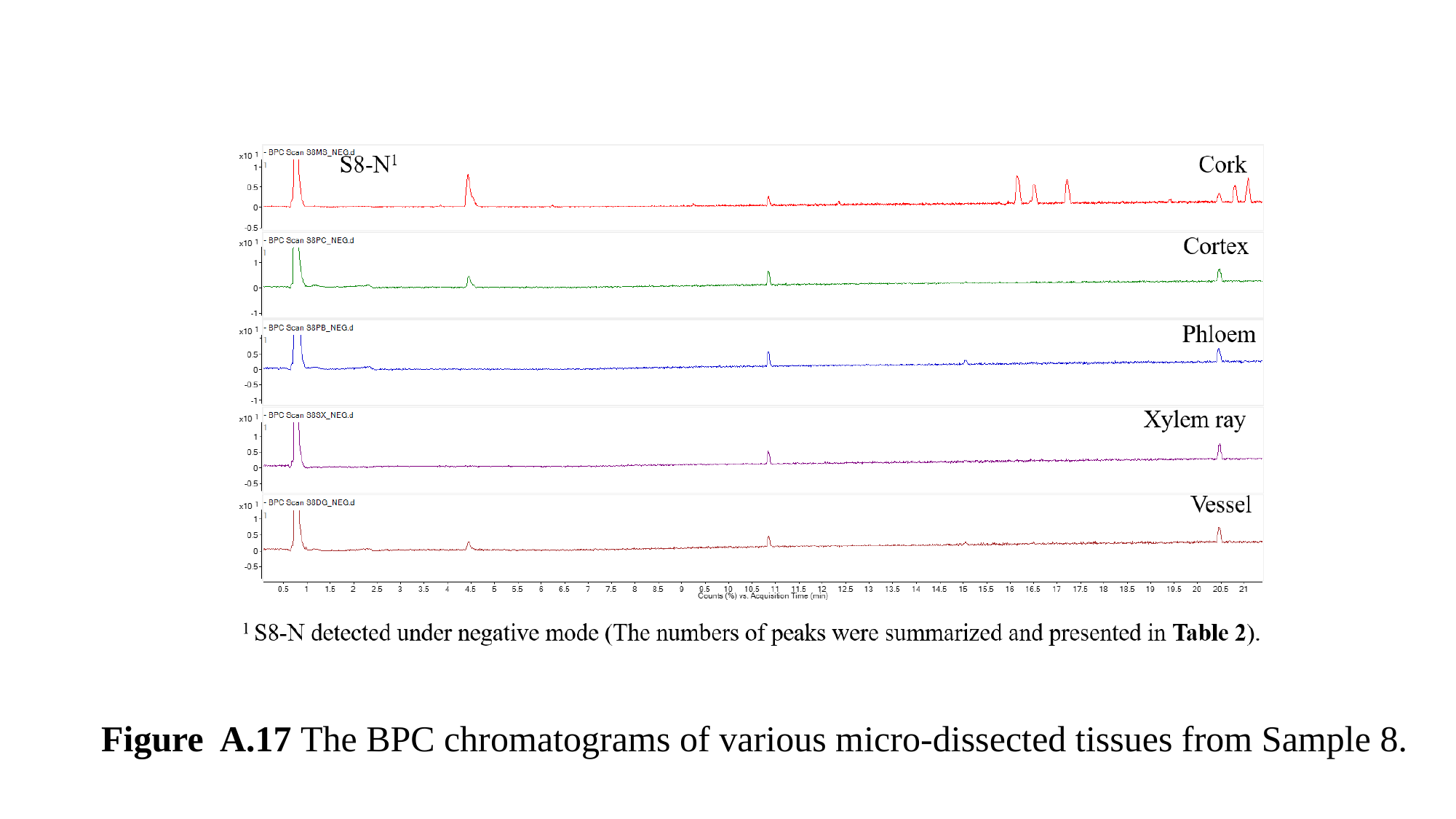

# Figure A.17 The BPC chromatograms of various micro-dissected tissues from Sample 8.

## Slide 18
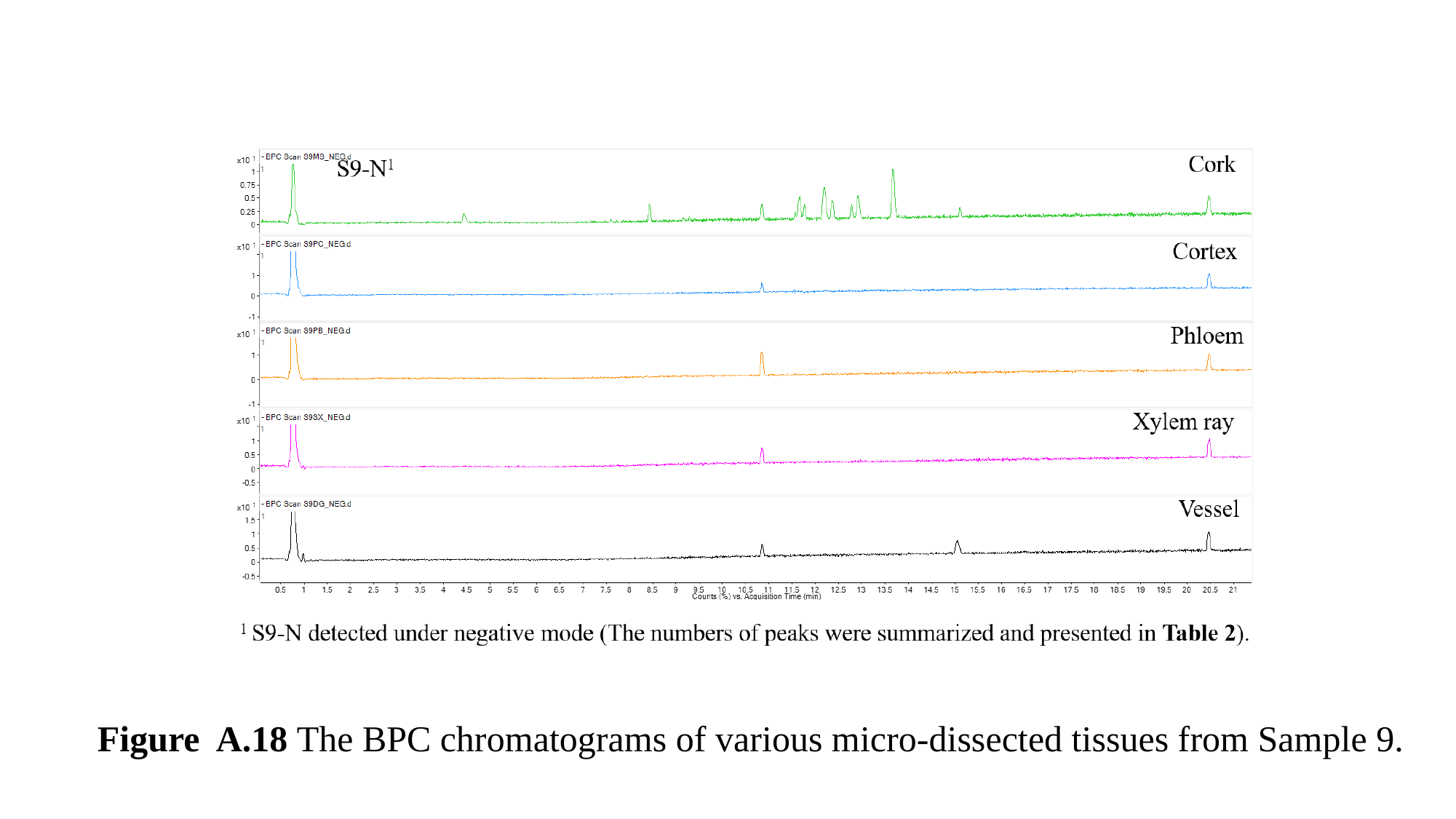

# Figure A.18 The BPC chromatograms of various micro-dissected tissues from Sample 9.
